# Rearing in Seawater Mesocosms Improves the Spawning Performance of Growth Hormone Transgenic and Wild-Type Coho Salmon

**DOI:** 10.1371/journal.pone.0105377

**Published:** 2014-08-18

**Authors:** Rosalind A. Leggatt, Tanya Hollo, Wendy E. Vandersteen, Kassandra McFarlane, Benjamin Goh, Joelle Prevost, Robert H. Devlin

**Affiliations:** Fisheries and Oceans Canada, West Vancouver Laboratories, West Vancouver, BC, Canada; Ghent University, Belgium

## Abstract

Growth hormone (GH) transgenes can significantly accelerate growth rates in fish and cause associated alterations to their physiology and behaviour. Concern exists regarding potential environmental risks of GH transgenic fish, should they enter natural ecosystems. In particular, whether they can reproduce and generate viable offspring under natural conditions is poorly understood. In previous studies, GH transgenic salmon grown under contained culture conditions had lower spawning behaviour and reproductive success relative to wild-type fish reared in nature. However, wild-type salmon cultured in equal conditions also had limited reproductive success. As such, whether decreased reproductive success of GH transgenic salmon is due to the action of the transgene or to secondary effects of culture (or a combination) has not been fully ascertained. Hence, salmon were reared in large (350,000 L), semi-natural, seawater tanks (termed mesocosms) designed to minimize effects of standard laboratory culture conditions, and the reproductive success of wild-type and GH transgenic coho salmon from mesocosms were compared with that of wild-type fish from nature. Mesocosm rearing partially restored spawning behaviour and success of wild-type fish relative to culture rearing, but remained lower overall than those reared in nature. GH transgenic salmon reared in the mesocosm had similar spawning behaviour and success as wild-type fish reared in the mesocosm when in full competition and without competition, but had lower success in male-only competition experiments. There was evidence of genotype×environmental interactions on spawning success, so that spawning success of transgenic fish, should they escape to natural systems in early life, cannot be predicted with low uncertainty. Under the present conditions, we found no evidence to support enhanced mating capabilities of GH transgenic coho salmon compared to wild-type salmon. However, it is clear that GH transgenic salmon are capable of successful spawning, and can reproduce with wild-type fish from natural systems.

## Introduction

Increasing growth rates of fish is one of the primary goals for advancement of aquaculture production. Selective breeding can increase growth rates over many generations and its use has been well established in aquaculture. In recent decades there has also been interest in using transgenic technologies to increase production. In particular, insertion of growth hormone (GH) transgenes has been demonstrated to dramatically increase growth rates in a number of fish species [1]–[7]. Atlantic salmon containing a chinook salmon growth hormone gene fused to an ocean pout antifreeze promoter is currently under consideration by the United States of America’s Food and Drug Administration for potential approval for human consumption [8]. If approved, it would become the first commercial transgenic animal used for human consumption. As transgenic technologies are relatively new, and phenotypic effects can be large, concern has been expressed regarding the potential environmental risks transgenic fish may pose to natural ecosystems. In particular, whether transgenic fish could breed with wild fish, thereby introducing the transgene to wild populations, or establish themselves in natural environments and potentially alter ecosystem food chains, is of concern [9]. The frequency of a transgene in populations will depend on both its rate of introduction and its effects on survival and reproduction (fitness) under different environmental conditions. GH transgenic fish can grow very fast, and in some cases can possess an adult body size greater than wild-type, which has been hypothesized to have the potential to provide a mating advantage [6], [10], [11]. Some phenotypes caused by GH transgenesis can also be advantageous under specific conditions (e.g. competitive foraging success [12]), whereas others cause negative fitness effects (e.g. reduced disease resistance and predator avoidance [13]–[15]). Previous modelling has found that a GH transgene conferring large effects on one of these fitness components could result in elimination or expansion of the transgene in populations [16]–[20]. Further, combinations of positive and negative pleiotropic effects (e.g. a mating advantage coupled with reduced viability) could theoretically cause population extinctions [11], [21], although genetic background of wild-type fish may provide counter-selection to restore population-level fitness [20]. Hence, understanding the ability of GH transgenic strains to reproduce is one critical component of estimating overall net effects on their fitness [22].

The reproductive ability of cultured GH transgenic coho and Atlantic salmon has been previously examined. Data thus far demonstrate that cultured GH transgenic salmon have the ability to show appropriate spawning behaviour and successfully spawn, but their reproductive success and level of behaviour is greatly decreased compared to wild-type salmon reared in nature [23]–[25]. In particular, when in competition with wild-type fish reared in nature, male and female GH transgenic coho salmon only contributed 3.9% to total F_1_ offspring generated in mixed-genotype spawning trials [24], and GH Atlantic and coho salmon males participated in 10% and 0% respectively of spawning events with nature-reared wild-type females [23], [25]. In paired trials, GH transgenic coho had 14–60% the success of nature-reared wild-type fish depending on the pairing [23], and male GH transgenic Atlantic salmon had 50% the spawning success of wild Atlantic salmon males [25]. In addition, female GH transgenic coho salmon performed fewer diggings and coverings in competition or in pairs [23], [24], and male GH transgenic coho and Atlantic salmon had greatly decreased aggressive behaviour and inconsistently decreased courtship behaviour compared to nature-reared wild-type fish [23]–[25].

The above information taken by itself suggests GH transgenic salmon have greatly decreased spawning abilities. However, where examined, there was also a very large effect of rearing conditions on wild-type salmon grown in the same laboratory culture conditions (as necessary for GH transgenic coho salmon) causing greatly reduced spawning success. In competitive trials with hatchery males reared in nature, cultured wild-type males contributed only 12.6% of offspring [24], and in paired spawning trials only 46–55% of cultured wild-type coho successfully spawned relative to nature-reared fish [23]. Further, cultured wild-type males and females showed decreased aggressive and courtship behaviours in competition with nature-reared fish [24], although they had equal male quivers and greater female digs in paired trials [23]. In addition, wild-type fish grown in equal culture conditions as GH transgenic fish had lower body weight and length compared to those raised in natural conditions from smolt, often had delayed maturation, and neither wild-type nor transgenic fish raised in culture had the mature red colouration or male kype and visible teeth of nature-reared fish [23]. This concurs with other studies that found juvenile [26]–[28] and lifetime [29]–[31] rearing in culture decreased spawning success in salmon. As such, whether the poor reproductive success of GH transgenic fish observed in previous studies is due to culture effects or to effects of the transgene (and their interactions) is unknown. Direct comparisons of transgenic and wild-type fish raised in equal conditions are limited and conflicting. Bessey et al. [23] found wild-type and GH transgenic coho males had equally poor success in competition for a single nature-raised female, while Moreau et al. [25] found GH transgenic Atlantic salmon mature male parr had lower spawning success than equally-raised wild-type mature male parr siblings in competition for a nature-reared female. Success of GH transgenic fish seems to be highly dependent on species and/or experimental conditions. For example, GH transgenic catfish and common carp were found to have approximately equal spawning success as wild-type fish [32], [33], GH transgenic medaka had increased [11], equal [22], or decreased [34] mating advantage over wild-type medaka depending on the study, conditions, and/or strain, and GH transgenic zebrafish had lower reproductive success than wild-type zebrafish [35]. As well, Pennington and Kapuscincki [36] found the reproductive success of male GH transgenic medaka relative to wild-type males was influenced by earlier rearing environments (i.e. level of food availability and presence of predators), suggesting genotype×environmental interactions influence the spawning success of GH transgenic fish.

Experiments in GH transgenic medaka found fitness data could accurately predicted the potential for an invading transgene to persist in a population, assuming fitness would not be influenced by the invaded ecosystem [34]. However, whether the relative capabilities for reproductive success between transgenic and wild-type salmon remain the same or differ when animals are reared in different environmental conditions (e.g. are there significant genotype-by-environment interactions affecting this phenotype?) is not known. Intentional release of GH transgenic fish into natural ecosystems to determine their spawning ability when raised in nature is not an acceptable experimental approach given the near impossibility of removing experimental animals to mitigate negative effects should they occur. In GH transgenic coho salmon, rearing in semi-natural contained stream conditions was found to dramatically restore wild-type phenotype and behaviour of juveniles [37]. In the present study, in an attempt to minimize the effects of culture on spawning success, we reared wild-type and GH transgenic coho salmon (*Oncorhynchus kisutch*) in large (350,000 L), semi-natural seawater tanks (hereafter termed mesocosms) from smolt to maturity. The mesocosms were designed to minimize culture effects by more closely mimicking nature than typical rearing in smaller tanks: natural water supply and lighting, low rearing densities, and minimum daily human-to-fish interactions. The purpose of our study was threefold: 1) to determine if and to what extent wild-type spawning success could be restored by mesocosm rearing, 2) to determine how GH transgenic spawning success and behaviour compares to wild-type salmon after seawater rearing in the mesocosm, and 3) to determine if the spawning success of GH transgenic salmon in nature can be extrapolated from this and previous data (i.e. are there genotype-by-environmental interactions in spawning success of GH transgenic and wild-type fish). For this we compared the spawning success and behaviour of mesocosm-reared transgenic and wild-type fish, as well as nature-reared wild-type fish, in three experimental conditions: as spawning male-female pairs (No Competition), with mixed male and female fish together competing for spawning sites and mates (Full Competition), and with two male types competing for wild-type nature-reared female mates (High Male Competition).

## Materials and Methods

### Experimental Animals

Spawning experiments took place at Fisheries and Oceans Canada’s Centre for Aquaculture and Environmental Research (CAER), West Vancouver, BC, Canada (49°20′N, 123°14′W), under requirements established by the Canadian Council for Animal Care. Approval and permits for these experiments were granted by the Pacific Region Animal Care Committee (Permit numbers: 09-009, 10-016, 11-016). All fish were from, or derived from, the sea-ranched Chehalis River hatchery population located in Southwestern British Columbia. Three main groups of fish were examined: GH transgenic fish raised in the mesocosm from smolt (termed T Mesocosm fish), wild-type fish raised in the mesocosm from smolt (NT Mesocosm fish), and wild-type fish raised in nature from smolt (NT Nature fish). NT Nature fish were hatchery-raised as freshwater juveniles at the Chehalis River Enhancement Facility, Agassiz, BC, Canada (49°16′N, 121°15′W, operated by and under authority from Fisheries and Oceans Canada), released to a natural river as smolts, and re-caught as mature salmon returning to the hatchery area to spawn at age 3 yrs. Transfers of fish from the Chehalis River Enhancement Facility to CAER were conducted under permits from Pacific Region Introductions and Transfers Committee (ITC), which is composed of authorities from Fisheries and Oceans Canada and the British Columbia Ministry of the Environment. NT Mesocosm fish were fish raised to smolt in hatchery conditions at CAER (2012) or a mix of those with fish raised in freshwater under hatchery conditions at the Chehalis River Hatchery (2009, 2010, 2013), followed by rearing in the seawater mesocosms until maturation at age 3 yrs. T Mesocosm fish were coho salmon hemizygous for the OnMTGH1 transgene (see [3], [38] for details), produced by crosses of wild-type Chehalis coho with strain M77 coho hemizygous or homozygous for the OnMTGH1 transgene. T Mesocosm fish were raised in hatchery conditions at CAER under one of two juvenile feeding level regimes: 1) For the Full Competition experiments, T fish were fed to satiation as juveniles to achieve accelerated growth and maturity at two-years old (these fish acquired smolt status in their first year and were transitioned to seawater in late summer along with their NT counterparts that were one year older), and 2) For the High Male Competition and No Competition experiments, T fish were pair fed a ration restricted to that of the NT fish during the juvenile phase (freshwater period prior to smolt) in order to prevent accelerated growth during this stage. These latter transgenic fish reach maturity at the normal three years of age rather than two years for satiated transgenic salmon. These T and their NT counterparts were transitioned to seawater in late spring before transfer to a mesocosm, closer to the normal smolt time for coho salmon. In one year (2010 spawning year, see Table 1 for relevant arenas), both NT and T Mesocosm fish were temporarily held in 12,800 L or 4000 L seawater tanks for 6 months prior to mesocosm entry (due to facility and operational issues). In Full Competition experiments, a fourth fish group was included: NT Culture fish were 3- to 4-year old fish raised in hatchery conditions at CAER to the smolt stage, then reared in standard culture seawater-fed tanks (4000 L) from smolt until maturity. Growth and survival during seawater mesocosm rearing are to be reported elsewhere.

**Table 1:** Experimental design of spawning experiments.

Experiment	Trial	n^1^
*I. No Competition (conducted in 2012)*		
	NT Mesocosm ♀×NT Mesocosm ♂	n = 7
	T Mesocosm ♀×T Mesocosm ♂	n = 7
	NT Nature ♀×NT Nature ♂	n = 7
	NT Nature ♀×NT Mesocosm ♂	n = 7
	NT Mesocosm ♀×NT Nature ♂	n = 7
	NT Nature ♀×T Mesocosm ♂	n = 8
	T Mesocosm ♀×NT Nature ♂	n = 8
*II. Full Competition (conducted in 2009, 2010, 2013)*		
	i. NT Mesocosm + T Mesocosm	n = 4 (1 in 2009^2^, 1 in 2010, 2 in 2013)
	ii. NT Mesocosm + NT Nature	n = 4 (2 in 2009, 2 in 2010)
	iii. T Mesocosm + NT Nature	n = 2 (2 in 2013)
	iv. NT Mesocosm + NT Culture	n = 1 (1 in 2009)
*III. High Male Competition (conducted in 2012)*		
	i. NT Mesocosm ♂ vs. T Mesocosm ♂	n = 3
	ii. NT Mesocosm ♂ vs. NT Nature ♂	n = 3
	iii. T Mesocosm ♂ vs. NT Nature ♂	n = 2

Fish groups are wild-type (NT) or growth hormone transgenic (T) coho salmon reared from smolt in a 350,000 L seawater Mesocosm, in Nature, or in standard Culture (4000 L tank). **I.** No Competition experiments consisted of single male×female pairs in spawning channels for 48 h. **II.** Full Competition experiments consisted of mixed male and female fish of two fish groups, 4 fish per fish group and sex, for a total of 16 fish in each spawning arena. **III.** High Male Competition experiments consisted of 4 male fish each of two different fish groups, competing for 4 NT Nature females, for a total of 12 fish in each spawning arena. For experiments **I.** and **III.** transgenic fish were ration restricted as juveniles (age at maturity = 3 years), and all NT fish were reared as juveniles at CAER. For experiment **II.** transgenic fish were fully fed as juveniles (age at maturity = 2 years), and NT fish were a mix of fish reared as juvenile at CAER, and those reared as juveniles at the Chehalis Enhancement Facility. Age of NT fish at maturity was 3 years (Mesocosm and Nature-reared) or 3–4 years (Culture-reared).

1 Experimental unit for I = one spawning pair, experimental units for II and III = one arena.

2 Due to limited fish numbers, this arena consisted of 3 fish per genotype and sex for a total of 12 fish.

### Mesocosm Rearing

At the smolt stage, all fish to be raised in the mesocosms were implanted with a Passive Integrated Transponder (PIT) tag, fin-clipped for genotype confirmation (as per [24]), weighed and measured, transitioned to seawater over 8–10 days, and then transferred to the mesocosm for seawater rearing until maturity. Mesocosm tanks were semi-natural, circular, 12.2 m in diameter and 3 m deep, for a total volume of 350,000 L each. Mesocosm conditions were designed to minimize culture effects associated with typical rearing in smaller tanks. This was attempted by maintaining abiotic factors closer to natural than under standard culture conditions. Mesocosms were fed with ambient-temperature, sand-filtered seawater from Burrard Inlet, BC. Unidirectional water inflow maintained a constant current within the tank to stimulate continual swimming by the fish. Lighting was natural, filtered through a translucent white tent cover. The mesocosms were fitted with a 1 m high screen, which had a primary purpose of minimizing visual perception of humans by the fish within the mesocosm building. Antibiotics and vaccinations were not administered with the exception of a single dorsal sinus injection of 20 mg/kg oxytetracycline administered to one half the mesocosm-reared fish from the 2012 spawning experiment 10 months after mesocosm entry (as part of an examination of survival in the mesocosm, to be reported elsewhere). Fish in the mesocosms were reared at very low densities atypical of normal culture conditions, with a maximum density of 0.58–1.79 kg/m^3^ (2009: 0.58 kg/m^3^; 2010: 0.78 kg/m^3^, 2013: 1.72 kg/m^3^, 2012: 1.79 kg/m^3^), with much lower densities throughout most of the mesocosm culture. In contrast, density of NT Culture fish reared in the 4000 L tanks was 4.3 kg/m^3^ at maturity. Fish were hand fed 2 times per day to satiation with commercial salmonid feed (Skretting Canada) using size adjusted feeds appropriate for specific developmental stages. In addition, fish from spawning years 2009, 2010, and 2012 were supplemented with feed from an automatic feeder 5x/day.

Prior to all spawning experiments, fish were seine-netted out of the mesocosms, lightly anaesthetised with tricaine methanesulfonate (100 mg/L, buffered with 200 mg sodium bicarbonate/L), weight and length recorded, and near-mature fish sorted into one of the following containers fed with well water at 10°C: 1) an artificial spawning channel, 2) a 9000 L holding tank (Full and High Male Competition experiments), or 3) a 12,500 L holding tank (No Competition experiments). Fish were held in well water for a minimum of one week to acclimate. Mature and near-mature NT Nature fish were collected as needed from the Chehalis River Hatchery in late December through January and held in freshwater at the laboratory until the start of the spawning trials.

### Spawning Experiments

Three main experiments were performed with varying levels of competition (see Table 1 and descriptions below for details): I) a No Competition experiment with single-paired male and female fish to determine if NT and T Mesocosm fish were capable of spawning, could display appropriate spawning behaviour, and whether they could spawn with NT Nature fish, II) a Full Competition experiment (both sexes of two types of fish) to determine the spawning success and behaviour of NT and T Mesocosm fish in competition, and III) a High Male Competition experiment (two types of males paired with one type of female) to examine the spawning behaviour and success of male NT and T Mesocosm fish in competition for NT Nature females. Male success was examined in greater depth, as male spawning success has been previously shown to be affected to a greater degree by external and internal factors than for female fish [26], [27], [39]–[41].

#### I. No Competition Experiments

No Competition experiments (i.e. single male and female pairs) took place from January to February, 2012 (see Table 1 for details). Experiments were conducted in eight 4.3×0.9 m spawning channels with an approximate water depth of 30 cm (excluding gravel), contained within a tent building with a translucent white cover. One submersible pump per channel created a unidirectional flow that was maintained at approximately 14 cm/sec in the middle of the channel during spawning experiments. Gravel size of the spawning channels was a mix of 2.5 and 3.8 cm average diameter, and was set at an average depth of 13 cm. Video equipment linked to a computer with Milestone XProtect Video Recording Software was mounted above every two channels for continuous behaviour recording. Channels were shielded with blue tarps at the sides and ceiling to minimize glare off the water during video recording. One 60-watt bulb was placed on the ceiling at the front of every two channels for continual dim lighting, in addition to natural diurnal light filtered through the tent and tarps. Water supply to the channels was well water at 10°C.

Experimental fish were removed from the holding tank and assessed for maturity. Only those fish with soft abdomens that expelled eggs (females), or free-flowing milt (males) when lightly pressed were used. Each spawning trial was initiated by placing one male and one female fish in each channel. The pair remained in the channels undisturbed for 48 h, during which time behaviour was continually recorded by the digital video camera system. At the end of 48 h, fish were removed, lightly anaesthetized, and measured for weight and length.

Behaviour of each spawning pair recorded on video was assessed for 5 min, every two hours for 48 h or until one of the pair had died. Video was then screened to identify all spawning events over the entire 48 hr period. Time to first spawn was recorded for each successful pair. For each spawning event behaviour was assessed for 5 min each at 30 min, 20 min, and 10 min prior to spawn, at spawn (0 min), and 10 min and 20 min post-spawn. As well, whether egg and/or milt release were visible at spawn was recorded for each spawning event if visibility allowed. Behaviours recorded were as follows: total time male attended or pursued female per 5 min, total time female maintained position over a nest per 5 min, number of quivers or gapes by male and female, and number of digs and covers by female.

#### Artificial Spawning Channel for Full and High Male Competition Experiments

Full and High Male Competition experiments (see below) were conducted in an artificial spawning channel. Total spawning channel dimensions were 2.1×30.5 m, with water depth 25–50 cm deep (excluding gravel). Two external, variable speed pumps created unidirectional flow that was maintained at approximately 12 cm/sec during spawning trials. Gravel size of the spawning channel averaged 3.8 cm in diameter, and was set at an average depth of 15 cm. The spawning channel was located within a tent with a white translucent cover that allowed natural lighting to filter in. The channel was supplied with flow-through fresh well water at 10°C, with the exception of the Full Competition experiments in 2009 where the channel was supplied with ambient temperature creek water. The channel was divided into 4 spawning arenas of 7.3×2.1 m in size (Full Competition experiments), or 8 arenas of 3.7×2.1 m in size (High Male Competition experiments), resulting in approximately 1.9 m^2^ per spawning female. This density is within the range observed for coho salmon in nature [42], although was less than the average redd size for coho (2.8 m^2^, [43]), resulting in medium to high density of spawning females as defined by Fleming and Gross [26]. Removable screens of 2.4 cm^2^ square wire mesh divided the arenas during spawning, which were covered with fine mesh (1.5 mm^2^) when eggs were estimated to have reached the eyed stage, in order to retain emerging fry within their respective arenas. One edge of the channel was shielded by a dark blue tarp with several slits cut per arena to allow for behavioural observations while minimizing observer effects.

#### II. Full Competition Experiments

Full Competition experiments consisted of equal numbers of male and female fish from two different fish groups per arena. Four males and four females each of two groups (defined by genotype (T or NT) and rearing condition (Mesocosm, Nature, or Culture) of fish were place in arenas (for a total of 16 fish per arena), resulting in four potential types of matings (1^st^ Group ♀×1^st^ Group ♂, 1^st^ Group ♀×2^nd^ Group ♂, 2^nd^ Group ♀×1^st^ Group ♂, and 2^nd^ Group ♀×2^nd^ Group ♂). Full Competition experiments were divided into for different trials as outlined in Table 1.

Experimental animals were lightly anaesthetized, and maturity assessed as in No Competition experiments above. In 2013, NT Mesocosm fish had low overall growth rates, and thus in this year larger mature fish from the NT population were chosen. Otherwise fish were chosen at random from available mature fish. Weight and length of mature fish was recorded, a section of fin was removed and placed in 95% ethanol for pedigree analysis, and fish were tagged with a Petersen tag colour and number coded for fish type, sex, and individual. The day prior to the start of the trials the fish were sorted into their appropriate arenas, with a screen separating male and female fish. The trials were initiated by removing the separating screen. Behavioural observations were made for 5 min per arena conducted four times per day starting at 9 am, 11 am, 1 pm and 3 pm. Behaviours recorded were aggressive behaviours (chases, bites), courtship behaviours (males attending females, quivers and gapes by males and females, digs and covers by females, see [23] for details), and spawning occurrences (milt and egg releases). Coho salmon are a semelparous species (i.e. they die shortly after spawning), and fish were allowed to undergo natural spawning mortality in the stream. All deceased fish were removed once daily, identification taken, egg weight recorded and remaining egg number estimated for females, and testes condition recorded for males. Behavioural observations continued in each arena until 1 day after all of one sex of fish had died within the arena

Fecundity (total egg weight, and estimate of total egg number) was taken on any surplus NT Mesocosm, T Mesocosm, and NT Nature female fish to estimate expected egg mass of female fish used in the spawning trial. In the 2013 year, these data were also used to estimate the expected number of offspring produced per female, as well as an estimation of % offspring survival. This calculation was not included for 2009 and 2010 year classes due to incomplete data on spawning females used in these trials.

When fry first emerged, water velocity was decreased to approximately 4.7 cm/sec. Fry were fed 2–4 times a day commercial crumb or mash diet (Skretting Canada). Approximately 1 month after emergence, offspring were removed from arenas and euthanized by an overdose of anaesthetic (200 mg/L tricaine methanesulfonate, 400 mg/L sodium bicarbonate). Total offspring numbers per arena were assessed. In 2009 and 2010, a random subset of euthanized fry were bled (caudal sever) into microtitre plate wells containing 100 µL of 0.01 N NaOH, and the remaining fish stored in 95% ethanol. In 2013, all euthanized fry were placed in ethanol, then a random subset removed and tail fins placed in 0.01 N NaOH. Tissues in NaOH were heated to 99°C for 5 min to liberate DNA and denature nucleases in preparation for pedigree analysis.

#### III. High Male Competition Experiments for NT Nature Females

High Male Competition experiments (i.e. equal numbers of two types of male fish competing for limited number of NT Nature female fish) took place from January - February, 2012. Experimental animals were tagged as above in Full Competition experiments. To initiate the trials, four NT Nature females were place in arenas, followed by four males each of two different groups (4x ♂ 1^st^ Fish Group+4x ♂ 2^nd^ Fish Group+4x NT Wild ♀ = 12 fish total with a male:female ration of 2∶1). High Male Competition experiments were divided into three different trials as outlined in Table 1.

Behavioural observations and final fish processing were as described above for the Full Competition experiments. Offspring were sampled for pedigree analysis as in 2009 and 2010 Full Competition experiments above.

### Pedigree Analysis

Parentage of a random subset of offspring from each arena in Full Competition and High Male Competition experiments were determined by microsatellite analysis. Parent tissue DNA was extracted into distilled water using DNeasy Kits (Qiagen Inc., Germantown, MD), or by placing fin clip in 0.01 N NaOH and heating to 99°C for 5 min. Parents were screened to identify informative loci and unique microsatellite alleles. All primers and reagents were obtained from Life Technologies (Austin, TX)/Applied Biosystems (Foster City, CA). Three to five of the following microsatellites primers were used per arena: *one111* [44]; *ots101* [45]; *omm1008* [46]; *omm1128*, *omm1135* [47]; *omm1231*, *omm1270* [48]; *omm1322* [49]; *omm1399* [50]; *omm5007*, *omm5008*, *omm5030*, *omm5090*, *omm5092* [51]; *omm5132* [52]; and *ssa407* [53]. Forward primers were tagged with one of four fluorescent probes (6FAM, VIC, NED, PET), and reverse primers contained a 7 bp tail (GTGTCTT). Amplification of microsatellites was conducted via PCR reactions in 96-well plates using a GeneAmp PCR system 2720 thermal cycler (Applied Biosystems). PCR reactions contained either 0.3–0.5 µL extracted tissue in 0.01 N NaOH or 1 µL extracted DNA in water, and 10 µL reaction mix containing 1x PCR buffer, 0.2 mM dNTPs, 2 mM MgCl_2_, 1.2–1.5 µM each forward and reverse primers, and 0.05 U Taq. PCR reactions were as follows: 1 cycle of 95°C for 10 min (denaturing); 30 cycles of 94°C for 30 sec (denaturing), 48–62°C for 30 sec (annealing), 72°C for 1 min (extending); 1 cycle of 72°C for 7 min (extending). Annealing temperatures for primers were as follows: 48°C (*omm5090*), 54°C (*ots101*), 56°C (*one111*), 60°C (*omm5132*), 61°C (*ssa407*), 62°C (*omm5008*), and 58°C (all remaining primers). After amplification, 0.75 µL of PCR reactions were combined with 10 µL HiDi Formamide containing 0.35–0.5 µL GS-LIZ500 size standard in a 96-well plate. Samples were heated to 99°C for 3 min then immediately cooled on ice. The PCR products were then detected and sized using a 3130x Genetic Analyzer (Applied Biosystems). Parents of offspring were determined using WhichParent software (available at http://bml.ucdavis.edu/research/research-programs/conservation/salmon-research/salmon-genetics-software/).

### Statistical Analyses

Statistical comparisons among fish groups for most spawning behaviour and success variables were through 1-way ANOVA, followed by Student-Neuman-Keuls (SNK) *post-hoc* test, where spawning pairs (No Competition experiments) or arenas (Full and Male Competition experiments) were considered experimental units. For proportional data (e.g. proportion of a fish group that successfully spawned), data were arcsine transformed prior to analysis. If Normality or Equal Variance tests failed, data were ln, reciprocal, or square root transformed and reanalyzed. If transformation failed to bring about Normality and Equal Variance, data were analyzed by Kruskal-Wallis ANOVA on Ranks, followed by Dunn’s *post-hoc* test.

For variables where more than one factor was of interest, 2-way ANOVA’s were performed, with transformations as above where appropriate. Analyses by 2-way ANOVA included variables in the Full Competition experiments where effects of both sex and fish group were of interest (e.g. % of offspring that fish contributed to, aggressive behaviour, etc.), and behaviour measurements during spawning events in No Competition experiments where fish group and time were factors. Weight and length were analyzed with fish group and year as factors. In addition, whether there were significant Arena×Fish Group interactions on raw data in Full and Male Competition experiments was examined (i.e. did the relative behaviour and success of fish groups differ among arenas). In the results, Arena effect is only addressed if there were significant Arena×Fish Group interactions.

In No Competition experiments, proportion analysis was done by Chi-squared analysis, or where values were small by Fisher-Exact test. In Full Competition experiments, whether there was significant assortative mating for each arena was analyzed by 2×2 contingency analysis with Yates correction [28]. In Full and High Male Competition experiments, whether there was significant influence of fish length on spawning success (number of offspring or number of mates per fish) was determined by linear regression.

In T Mesocosm + NT Nature Full and High Male Competition trials, n = 2 arenas resulted in poor statistical power for between-arena comparisons. In these trials, there were no significant Arena×Fish Group interactions (p-values for behaviour and spawning success ranged from 0.101 to 0.882). Therefore, data from the two arenas were combined and analyzed with individual fish as experimental units. A statement of difference for each comparison is made in the text only if p<0.05. Statistical analyses were performed using SigmaStat (San Jose, CA). Data are presented as mean ± standard error of the mean.

## Results

### Fish Size and Morphology

**Figure 1:**
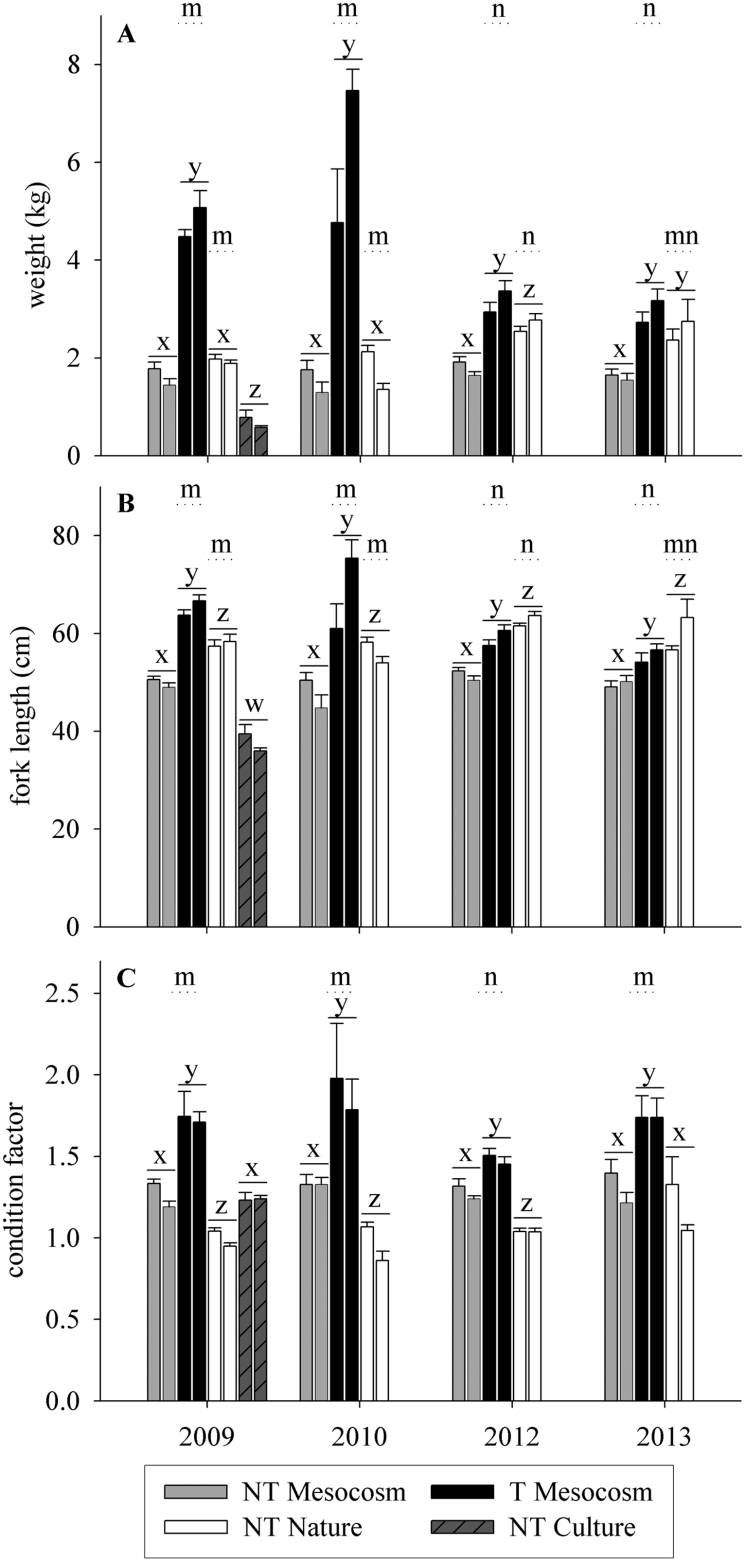
Size of fish used in spawning trials. **A)** Weight (kg), **B)** fork length (cm), and **C)** condition factor. Fish groups are wild-type fish raised in the mesocosm (NT Mesocosm), in natural conditions (NT Nature), or standard culture conditions (NT Culture) from smolt, and GH transgenic fish raised in the mesocosm from smolt (T Mesocosm). Data are given as female then male for each fish type. Fish in years 2009, 2010, and 2013 were used in the Full Competition experiments, measurements taken before entry in the spawning channel, and transgenic fish were fully fed as juveniles. Fish in year 2012 were used in the High Male Competition and No Competition experiments, measurements taken at time of mortality or post-trial respectively, and transgenic fish were fed a wild-type ration as juveniles. w,x,y,z over a solid line indicates differences among fish groups within years summed over sex, and m,n over a dotted line indicates differences within fish groups among years, summed over sex.

Fish weight, length, and condition factor (CF) used in the spawning trials are given in Figure 1A–C. In all years transgenic (T) Mesocosm fish were greater in weight and length than wild-type (NT) Mesocosm fish (p<0.001). NT Mesocosm fish were similar in weight to NT Nature fish in 2009 and 2010, but smaller in weight in 2012 and 2013 (p<0.001). This was primarily due to increased weight of NT Nature fish in later years (p<0.001), as the weight of NT Mesocosm fish was not significantly different between years (p = 0.406). In all years NT Mesocosm fish were shorter in length than NT Nature fish (p<0.001). T Mesocosm fish were significantly larger in weight than NT Nature fish in 2009, 2010, and 2012 (p<0.001) but similar in weight in 2013. T Mesocosm fish were larger in length in 2009 and 2010, and smaller in length than NT Nature fish in 2012 and 2013 (p<0.001). The changing relative sizes of T Mesocosm and NT Nature fish were due to both an increase in NT Nature fish size and a decrease in T Mesocosm size in later years. In general, CF was ranked in order of T Mesocosm > NT Mesocosm > NT Nature, although differences were not significant in 2013 between NT Mesocosm and NT Nature fish. In 2009, NT Culture fish were considerably smaller than all other groups in both weight and length (p<0.001), but only differed in CF from NT Nature fish.

**Figure 2:**
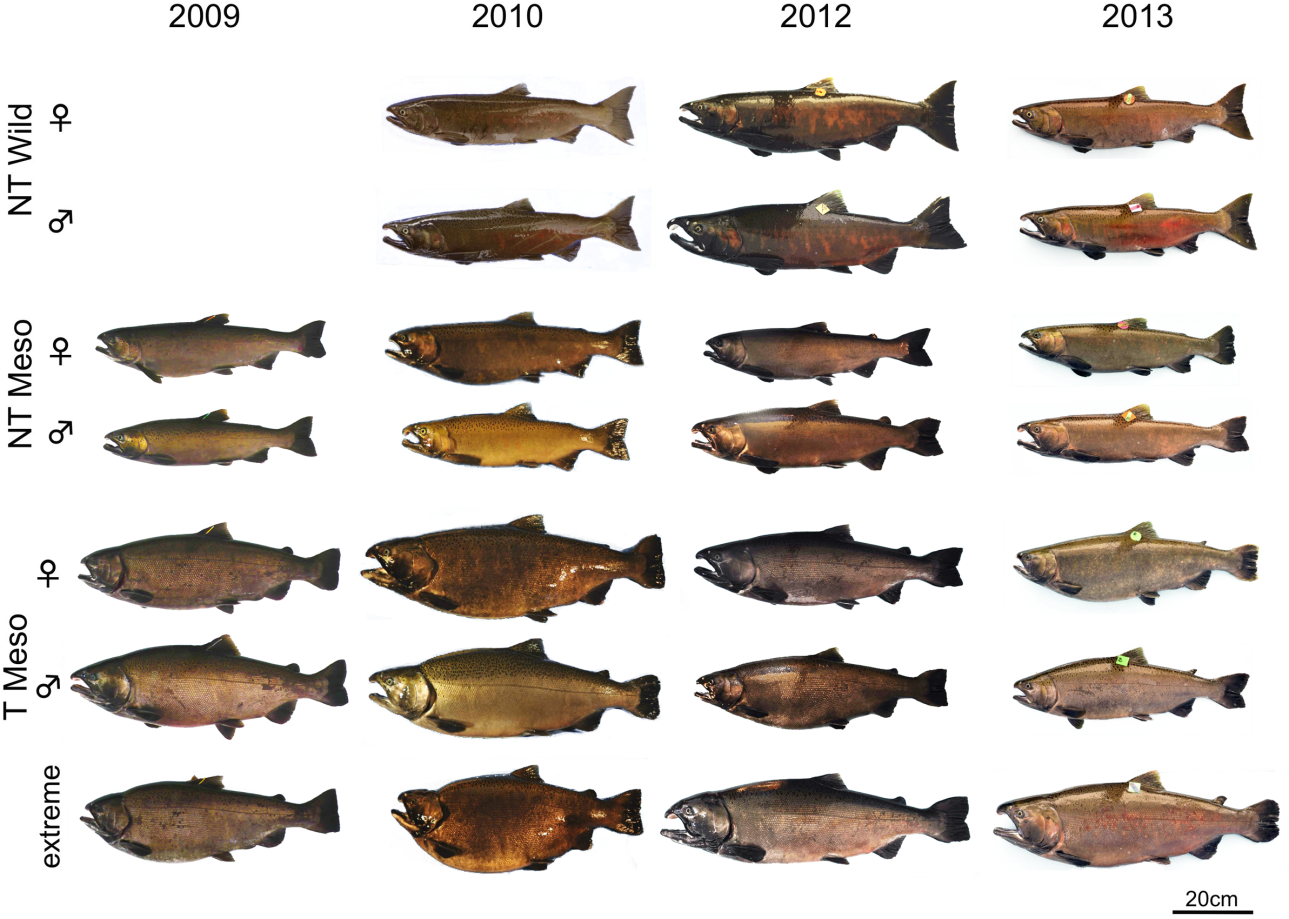
Representative morphology of fish used in spawning trials. Fish groups are wild-type fish raised in natural conditions from smolt (NT Nature), or in the mesocosm from smolt (NT Meso), and GH transgenic fish raised in the mesocosm from smolt (T Meso). Representative fish expressing the most extreme morphology of T Meso fish are also given in each year. In years 2009, 2010, and 2013, transgenic fish were fully fed as juveniles, and in 2012 were fed a ration restricted to that of NT fish as juveniles. Morphology of NT Nature fish for 2009 spawning year was not available.

Spawning morphology of representative fish used in the experiments are given in Figure 2. NT Nature fish displayed typical spawning morphology of coho salmon: red colouration of sides in males and females, and elongated and hooked jaw and humped back in males. Mesocosm-raised fish developed some of the red colouration observed in NT Nature fish, although colouration tended to be dark brown rather than red in most fish. Male NT Mesocosm and T Mesocosm fish developed the hooked jaw (kype) of NT Nature males, although without the elongated jaw observed in nature-reared fish. Minor hump development associated with sexual maturation was observed in only some NT Mesocosm males, and not observed in T Mesocosm males. Mesocosm-raised fish tended to have less developed or more eroded tails than NT Nature fish, particularly T Mesocosm fish. While NT Mesocosm fish approached the fusiform shape of NT Nature fish, T Mesocosm fish tended to be deeper bodied than both groups of fish. In 2010, the deeper body shape of T Mesocosm fish was extremely exacerbated in a few fish (Figure 2, extreme). In addition, excessive cranial growth was observed in some T Mesocosm fish.

### I. No Competition Experiments

To determine the influence of GH transgenesis and mesocosm culture on the ability of salmon to spawn with a member of their same group and with NT Nature fish, and to display appropriate spawning behaviour in the absence of competition, we examined spawning success and behaviour of T Mesocosm, NT Mesocosm, and NT Nature fish in No Competition (paired) experiments over 48 h.

#### Ia. Spawning Success

**Figure 3:**
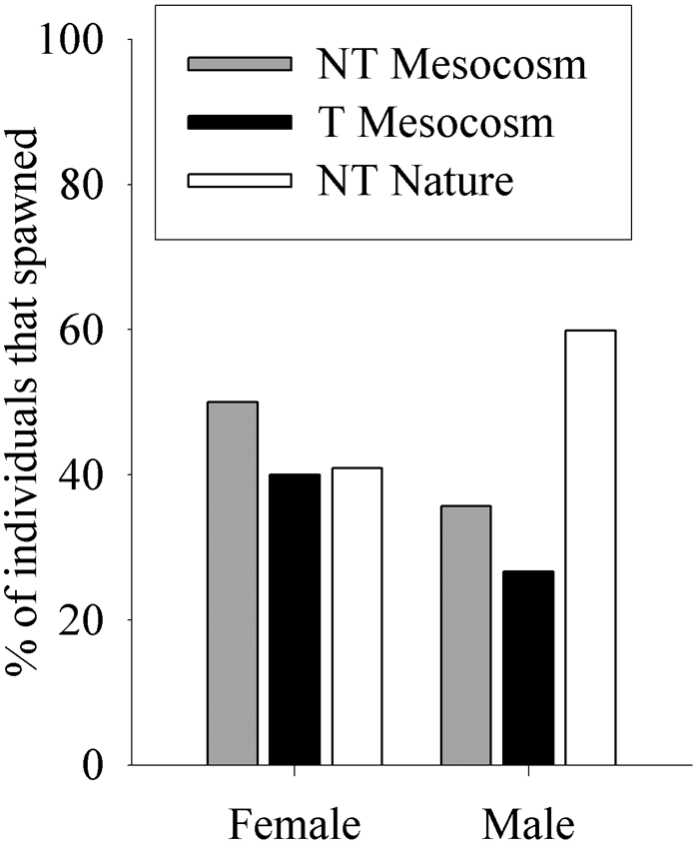
Spawning success (% that spawned) of fish groups in No Competition experiments. Fish groups are wild-type (NT) and growth hormone transgenic (T) coho salmon raised from smolt in a Mesocosm or in natural (Nature) conditions, summed over male or female fish.

There were no significant differences in the proportion of pairs that spawned in any of the crosses examined (p = 0.419, Table 2), although these results should be interpreted with caution as the power of the test for this comparison was low (0.396). When the percent of fish that spawned was summed over male partner, there were no significant differences in spawning success of females (p = 0.830, power = 0.077, Figure 3). When the percent of fish that spawned was summed over female partner, NT Nature males had 2.2-fold greater spawning success than T Mesocosm males and 1.7-fold greater spawning success than NT Mesocosm males, although these differences were not significant (p = 0.119, Figure 3). However, these results also should be interpreted with caution due to low power of the test for this comparison (0.425). The time to first spawn did not differ between different types of spawning pairs (p = 0.764, see Table 2), female type summed over male (p = 0.286), or male type summed over female (p = 0.684). The percent of spawning events where egg release was visible did not differ among spawning pairs (p = 0.533, Table 2) or among female type summed over male partner (p = 0.757, average 46.7% with visible eggs). However, the percent of spawning events where milt release was visible did differ among spawning pairs (p = 0.046, Table 2), where NT Nature female×NT Nature male and NT Mesocosm female×NT Nature male pairs had greater percent of spawning events with visible milt than NT Nature female×NT Mesocosm male. As well, when male type summed over female partner was examined, NT Nature males had approximately 2-fold more events with visible milt release than NT Mesocosm males (88% and 44.4% respectively, p = 0.017). T Mesocosm males had similar percent of events with visible milt release as NT Mesocosm males (50%), but did not differ significantly from NT Nature males (p = 0.127).

**Table 2:** Number of crosses used, spawning success (% of pairs that spawned), and % of spawning events where egg or milt release is visible in No Competition experiments of NT Mesocosm, T Mesocosm, and NT Nature coho salmon.

Cross	% of pairs thatspawned^1^	Time (h) to firstspawn	% with visibleegg release	% with visible miltrelease
*NT Mesocosm ♀*×*NT Mesocosm* ♂	42.9 (41.7)	23.3±7.5	33.3	66.7^ab^
*T Mesocosm* ♀×*T Mesocosm* ♂	28.6 (20)	26.6±17.3	50	50^ab^
*NT Nature* ♀×*NT Nature* ♂	71.4 (90.9)	11.8±4.0	31.3	87.5^a^
*NT Nature* ♀×*NT Mesocosm* ♂	28.6 (50)	13.4±5.9	66.7	0^b^
*NT Mesocosm* ♀×*NT Nature* ♂	57.1 (n/a)	21.2±6.4	50	100^a^
*NT Nature* ♀×*T Mesocosm* ♂	25.0 (55.6)	17.9±7.5	100	50^ab^
*T Mesocosm* ♀×*NT Nature* ♂	50 (12.5)	18.0±5.8	60	80^ab^

NT = wild-type coho salmon, T = growth hormone transgenic coho salmon, Mesocosm = fish were raised from smolt in a mesocosm, Nature = fish were raised from smolt in natural conditions.

1 number in parentheses is equivalent % of pairs that spawned in cultured fish from [23].

a,b indicates significant differences between pairs, p<0.05.

#### Ib. Behaviour at Perispawn

**Figure 4:**
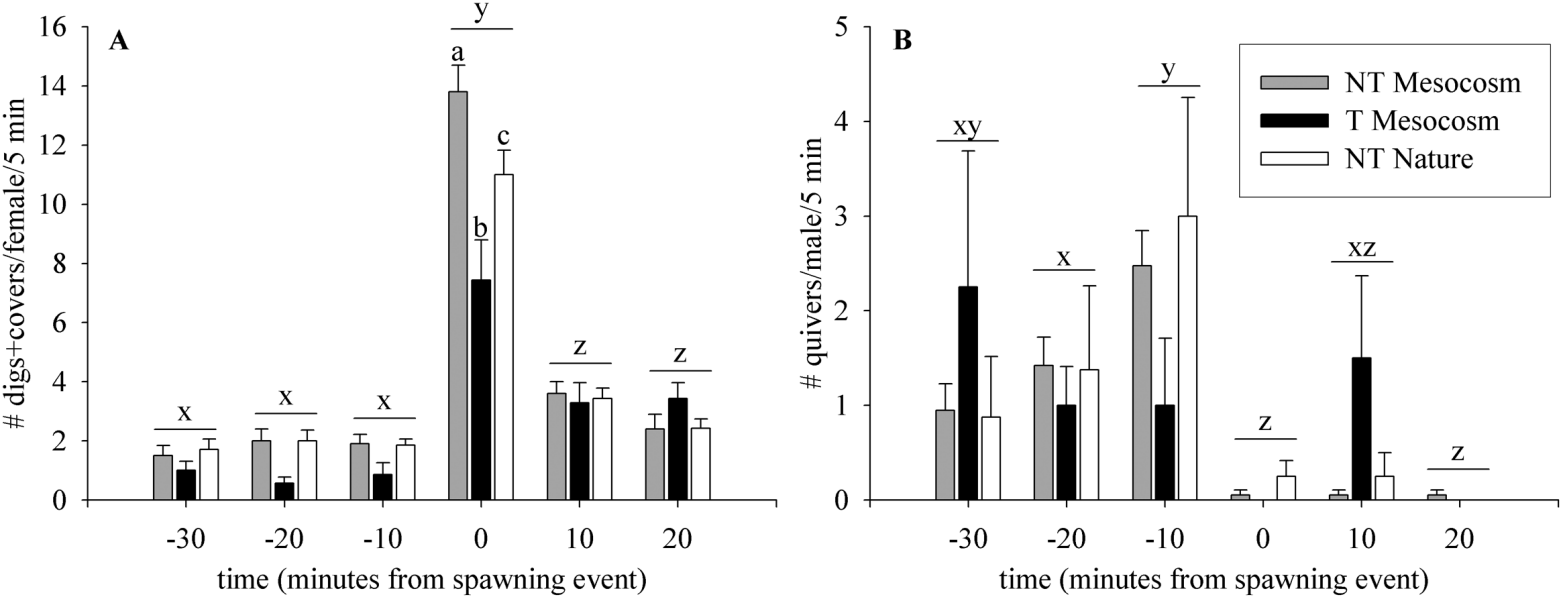
Behaviour of fish groups during spawning events in No Competition experiments. Fish groups are wild-type (NT) and growth hormone transgenic (T) coho salmon raised from smolt in a Mesocosm or in natural (Nature) conditions. **A)** Number of digs by females during 5 min intervals and **B)** number of quivers by males during 5 min intervals measured −30, −20, −10, 0, +10, and +20 min from spawning events. Data are given as means over females or males ± standard error of the mean. Significant differences between groups within time period are indicated by (a,b,c), and significant differences between time points summed over groups are indicated by (x,y,z), p<0.05.

During spawning events, there were no differences between fish types in any observed behaviour when summed over 30 min (Table 3). When individual times surrounding the spawning events were examined, T Mesocosm females performed approximately 30% less digs than NT Nature or NT Mesocosm fish overall prior to the spawning event when summed over male fish (p<0.001, Figure 4A), although did not differ at individual time points. As well, immediately after spawn all three groups differed in number of covers by females where NT Mesocosm > NT Nature > T Mesocosm (p = 0.001). There were no significant differences between fish groups in the number of quivers performed by males around the spawning event (p = 0.829, Figure 4B). However, both NT Nature and NT Mesocosm males had typical pattern of quivers reported for other salmonids (e.g. [54]), where number of quivers increased with time up to the spawning event, and then greatly decreased after the spawning event. This pattern of quivers over time was not observed in T Mesocosm males, who decreased quivers over time on average up to the spawning event, and had a high number of quivers 10 min post-spawn (see Figure 4B).

**Table 3:** Spawning behaviour during No Competition experiments summed over 30 min measured in 5 min intervals at −30, −20, −10, 0, +10, +20 min from a spawning event.

Cross	Time that ♀Maintainsnest (min)	Time that ♂attends ♀(min)	Number ofdigs by ♀	Number ofquivers by ♂
*NT Nature* ♀×*NT Nature* ♂	25.4±1.7	26.7±0.8	25.2±1.6	4.6±0.9
*NT Mesocosm ♀*×*NT Mesocosm* ♂	24.9±1.5	24.4±2.2	23.7±1.2	8.8±4.3
*T Mesocosm* ♀×*T Mesocosm* ♂	25.7±3.1	25.9±0.9	18.0±1.0	7.0±3.0
*NT Nature* ♀×*NT Mesocosm* ♂	24.3±0.8	28.5±1.5	21.5±5.5	1.5±0.5
*NT Mesocosm* ♀×*NT Nature* ♂	29.3±0.7	25.6±0.7	27.1±1.7	5.1±1.3
*NT Nature* ♀×*T Mesocosm* ♂	24.0±0.7	27.7±1.6	16.5±0.5	4.5±2.5
*T Mesocosm* ♀×*NT Nature* ♂	26.8±1.8	23.8±2.9	14.9±4.4	5.4±1.3

NT = wild-type coho salmon, T = growth hormone transgenic coho salmon, Mesocosm = fish were raised from smolt in a mesocosm, Nature = fish were raised from smolt in natural conditions.

#### Ic. Courtship Behaviour over 48 h

Different crosses did not differ in the overall time females maintained position over their nests (p = 0.641), the number of locations that females maintained nests (p = 0.457), or number of male quivers performed (p = 0.129, Table 4) per 5 min interval averaged every 2 h for 48 h. When fish were paired with a member of their same group, NT Nature females performed 4.6-fold and 3.4-fold more digs and covers than NT Mesocosm or T Mesocosm females respectively (p = 0.013), and NT Nature males spent 2.4-fold more time attending females than NT Mesocosm or T Mesocosm males (p = 0.018, see Table 4).

**Table 4:** Courtship and spawning behaviour during No Competition experiments over 5 min, average every 2 h over 48 h or until one fish died.

Cross	Time (sec) that♀ maintains nest	Time (sec) that♂ attends ♀	Number ofdigs by ♀	Number ofquivers by ♂	Number of nests ♀maintains over 48 h
*NT Nature* ♀×*NT Nature* ♂	105.8±32.8	181.2±26.6^a^	1.39±0.37^a^	0.06±0.06	2.0±0.5
*NT Mesocosm ♀*×*NT Mesocosm* ♂	56.1±20.0	79.0±21.1^b^	0.31±0.10^b^	0.07±0.03	1.3±0.2
*T Mesocosm* ♀×*T Mesocosm* ♂	76.5±20.7	72.4±30.2^b^	0.41±0.13^b^	0.01±0.01	1.4±0.3
*NT Nature* ♀×*NT Mesocosm* ♂	81.9±24.4	131.9±37.6	0.67±0.20	0.07±0.04	1.3±0.2
*NT Mesocosm* ♀×*NT Nature* ♂	82.4±26.6	131.2±35.8	0.83±0.26	0	1.1±0.3
*NT Nature* ♀×*T Mesocosm* ♂	68.1±5.0	108.6±23.6	0.31±0.05	0	1.8±0.2
*T Mesocosm* ♀×*NT Nature* ♂	48.6±22.0	121.0±26.7	0.35±0.23	0.06±0.03	1.1±0.4

NT = wild-type coho salmon, T = growth hormone transgenic coho salmon, Mesocosm = fish were raised from smolt in a mesocosm, Nature = fish were raised from smolt in natural conditions.

a,b indicates significant differences between pairs when fish are paired with a member of their same group, p<0.05.

### II. Full Competition Experiments

To determine whether GH transgenesis and/or mesocosm rearing influences the spawning success of salmon during competition, we compared the spawning success of T Mesocosm, NT Mesocosm, and NT Nature fish in mixed male and female competitions (Full Competition).

#### IIa. Spawning Success

**Figure 5:**
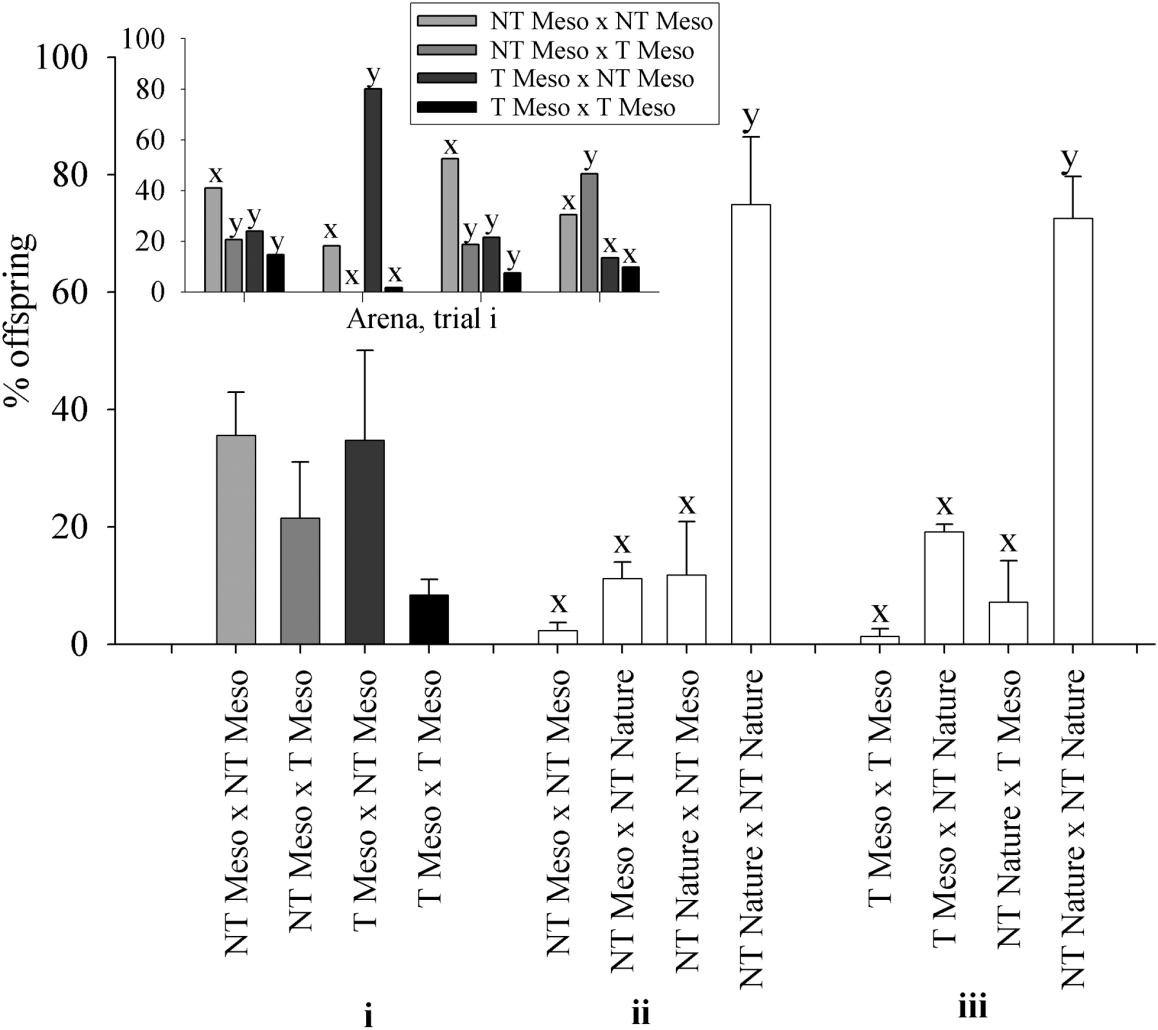
Percent of offspring produced by different matings during Full Competition experiments. Fish groups are wild-type (NT) and growth hormone transgenic (T) coho salmon raised in a mesocosm (Meso) or natural (Nature) conditions from smolt. Each spawning arena contained two groups of fish, four fish of each sex/group, competing for spawning sites and mates. Matings (bars) are given as Female×Male parent. Trials were: **i)** *Effect of transgene:* NT Mesocosm + T Mesocosm (n = 4 arenas), **ii)** *Effect of mesocosm rearing:* NT Mesocosm + NT Nature (n = 4 arenas), and **iii)** *Effect of transgene and mesocosm rearing:* T Mesocosm + NT Nature (n = 2 arenas). For trial i, the **Insert** provides % offspring for individual arenas. Significant differences among matings within trials or arenas are indicated by letters (x,y), p<0.05.

**Figure 6:**
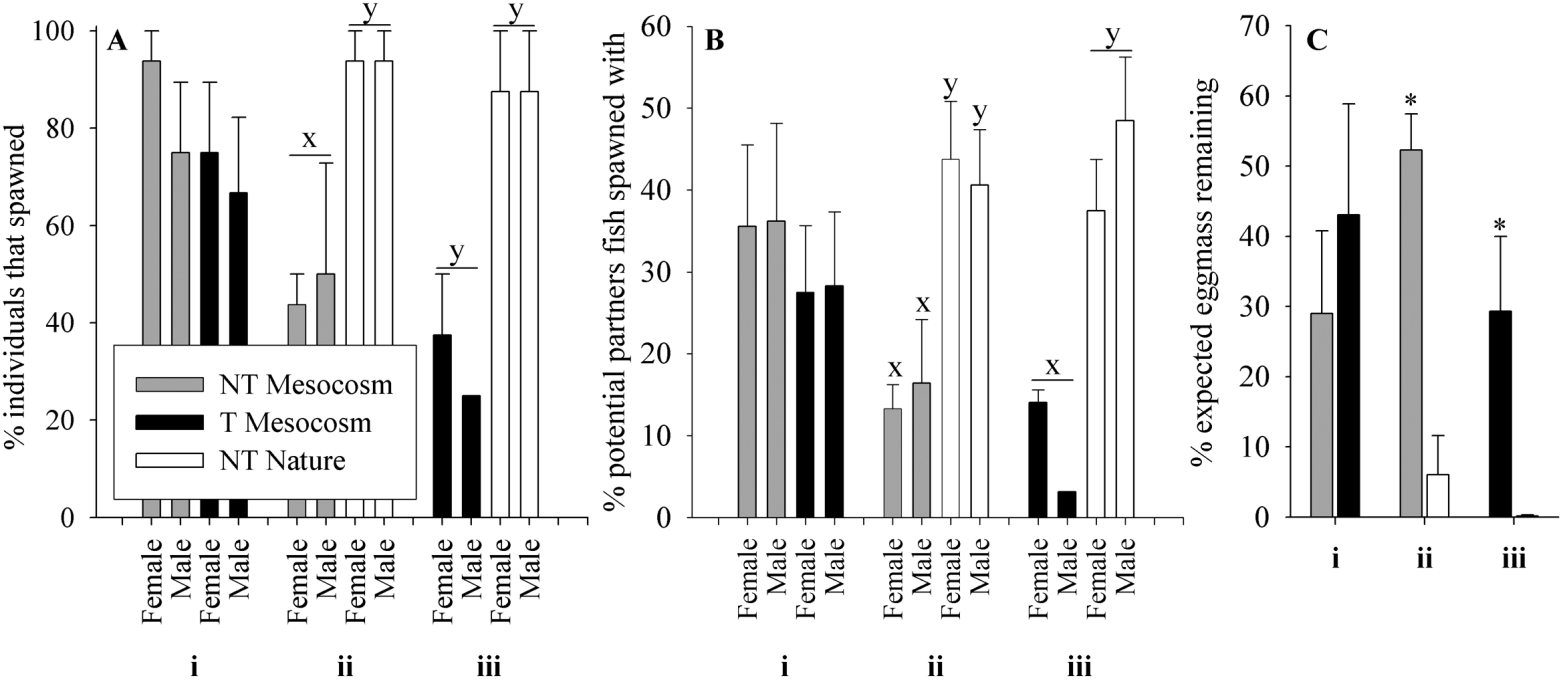
Mating success of fish groups in Full Competition experiments. Fish groups are wild-type (NT) and growth hormone transgenic (T) coho salmon raised in a Mesocosm or natural (Nature) conditions from smolt. Spawning arenas contained two groups of fish, four fish of each sex/group, competing for spawning sites and mates. Trials were: **i)** *Effect of transgene:* NT Mesocosm + T Mesocosm (n = 4 arenas), **ii)** *Effect of mesocosm rearing:* NT Mesocosm + NT Nature (n = 4 arenas), and **iii)** *Effect of transgene and mesocosm rearing:* T Mesocosm + NT Nature (n = 2 arenas). **A)** % of individuals that spawned, **B)** % of available partners individual fish spawned with, **C)** % of expected egg mass remaining at time of spawning mortality. Data are given as means over arenas ± standard error of the mean. Significant differences within trial type between groups/sex are indicated by letters (x,y), p<0.05.

*Effect of Transgene (NT Mesocosm + T Mesocosm):* Refer to section i in Figure panels for relevant data. All groups of fish showed the ability to mate, however the pattern of percent offspring from matings in NT Mesocosm + T Mesocosm spawning trials differed greatly between years and arenas (Figure 5 Insert). In two arenas, NT Mesocosm female×NT Mesocosm male matings produced the most offspring, while in the other two arenas, either T Mesocosm female×NT Mesocosm male or NT Mesocosm female×T Mesocosm male matings produced the most offspring. In all years T Mesocosm female×T Mesocosm male matings produced the fewest offspring, but not by a significant margin. When data from all arenas were averaged, there were no significant differences in percent offspring produced by the different matings (p = 0.218, Figure 5i). In addition, there were no differences between T Mesocosm and NT Mesocosm fish in the % of individuals that spawned (p = 0.382, Figure 6Ai) or the percent of total available partners that individual fish spawned with (p = 0.801, Figure 6Bi). Neither offspring number nor percent of total available partners individual fish spawned with significantly correlated with fish length in any group (p = 0.400 to 0.947). Contingency tables to analyze for assortative mating were not significant in any arena for this or any other Full Competition trial (ii, iii, and iv below, p = 0.492 to 0.876). The percent expected egg mass remaining in NT Mesocosm and T Mesocosm females post spawning mortality did not differ (p = 0.383, Figure 6Ci). Of the fifteen NT Mesocosm females used in total, all but one contributed offspring to the spawning trials, and this one female had an egg mass at mortality that was consistent with that of an unspawned NT Mesocosm female (predicted from fecundity-body size relationships, data not shown). Of the fifteen T Mesocosm females used, four did not contribute offspring to the spawning trials. Of these, three females had smaller egg mass than expected for an unspawned T Mesocosm female (0–67% of expected mass, average 24.2±21.6%).

*Effect of Mesocosm Relative to Nature Rearing (NT Mesocosm + NT Nature)*: Refer to section ii in Figure panels for relevant data. In three of four arenas, offspring from NT Nature female×NT Nature male matings far surpassed all other matings in number (82–90% total offspring), while in the remaining arena there were similar offspring numbers from NT Nature female×NT Nature male and NT Nature female×NT Mesocosm males matings (40% and 37% total offspring respectively). It should be noted that in this latter arena all NT Nature males died early in the spawning trial, leaving only NT Mesocosm males available to mate in the later half of the trial. Overall, NT Nature female×NT Nature male matings accounted for the majority of offspring (74.8%, p<0.001, Figure 5ii), with other matings accounting for approximately 11% (NT Nature female×NT Mesocosm male, and NT Mesocosm female×NT Nature male) or 2.3% (NT Mesocosm female×NT Mesocosm male) of offspring. In addition, twice as many NT Nature fish spawned than NT Mesocosm fish (p = 0.003, see Figure 6Aii), and NT Nature fish had 35% more partners on average than NT Mesocosm fish (p = 0.009, see Figure 6Bii). There was a weak but significant positive correlation between fish length and number of offspring produced by NT Nature female fish (p = 0.010, R^2^ = 0.39), as well as between fish length and number of mates in NT Nature male and female fish (p = 0.024 R^2^ = 0.31, and p = 0.043 R^2^ = 0.26 respectively), but not for NT Mesocosm fish. NT Mesocosm females had 8.6-fold greater percent of expected egg mass remaining post spawning mortality (p = 0.015), and all but one NT Nature female had no or very few eggs remaining post spawning mortality (Figure 6Cii). Of the sixteen NT Mesocosm females used in total, nine did not contribute offspring to the spawning trial. Of these, six NT Mesocosm females had smaller egg mass at mortality than expected for unspawned NT Mesocosm females (2–79% of expected mass, average 51.1±10.7%). Of the sixteen NT Nature females used, all but one female contributed offspring to the spawning trial, and this one female had an egg mass at mortality consistent with what was expected for an unspawned NT Nature female fish.

*Effect of Transgene and Mesocosm Rearing (T Mesocosm + NT Nature)*: Refer to section iii in Figure panels for relevant data. In the two arenas examined, offspring from NT Nature female×NT Nature male matings accounted for the majority of offspring (p = 0.038, 72.5%), while other matings accounted for 19.1% (T Mesocosm female×NT Nature male), 14.2% (NT Nature female×T Mesocosm male), or 1.3% (T Mesocosm female×T Mesocosm male) of total offspring (Figure 5iii). In addition, 3 times as many NT Nature fish spawned than T Mesocosm fish (p = 0.007, Figure 6Aiii), and NT Nature fish had 5 times as many partners on average than T Mesocosm fish (p = 0.001, Figure 6Biii). There was a significant positive correlation between fish length and number of offspring for T Mesocosm female fish (p = 0.015, R^2^ = 0.66), but no other significant correlations between length and spawning success were noted. T Mesocosm females had more that 100-fold greater percent of expected egg mass remaining post spawning mortality than NT Nature females (p = 0.003), and all NT Nature females had no or very few eggs remaining post spawning mortality (Figure 6Ciii). Of the eight T Mesocosm female fish used, five did not contribute offspring to the spawning trial, and all of these five fish had lower egg mass at mortality than expected for an unspawned T Mesocosm female (28–72% of expected, average 55.1±8.2%). Of the eight NT Nature females used, all contributed offspring to the spawning trial.

*Effect of Mesocosm Rearing Relative to Standard Culture (NT Mesocosm + NT Culture)*: In the one arena examined, matings with NT Mesocosm paternal parents had 1.9-fold greater offspring numbers than those with NT Culture paternal parents (68.3% and 31.7% total offspring respectively, p<0.001), while matings with NT Culture maternal parents had 1.2x greater offspring numbers than those with NT Mesocosm maternal parents (54.3% and 45.7% total offspring respectively, p = 0.029). There was no difference between the two groups of fish in % of individuals that spawned (75% and 100% for NT Mesocosm and NT Culture respectively, p = 1.00) or % of total available partners individual fish spawned with (31.3% and 37.5% for NT Mesocosm and NT Culture respectively, p = 0.623). NT Mesocosm and NT Culture females had similar percent egg mass remaining at spawning mortality (29.2±19.6% and 29.0±18.0% respectively, p = 0.769). Of the four NT Mesocosm and four NT Culture females used, only one NT Mesocosm female did not contribute offspring to the trial. This one fish had lower egg mass at mortality than expected for an unspawned NT Mesocosm female (13.0% of expected).

#### IIb. Behaviour

**Figure 7:**
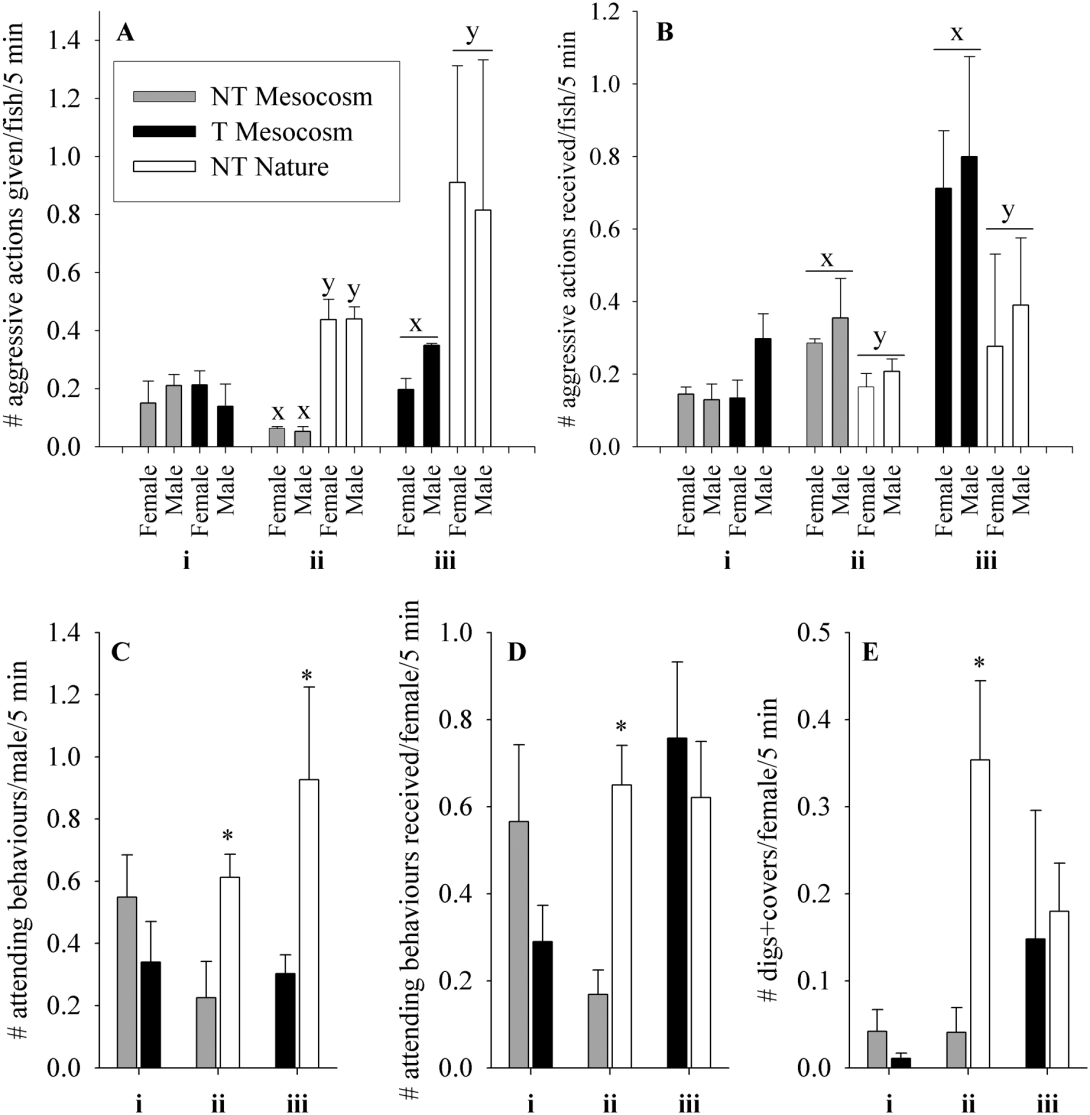
Spawning behaviour of fish groups during Full Competition experiments. Fish groups are wild-type (NT) and growth hormone transgenic (T) coho salmon raised in a Mesocosm or natural (Nature) conditions from smolt. Each spawning arena contained two groups of fish, four fish of each sex/group, competing for spawning sites and mates. Trials were: **i)** *Effect of transgene:* NT Mesocosm + T Mesocosm (n = 4 arenas), **ii)** *Effect of mesocosm rearing:* NT Mesocosm + NT Nature (n = 4 arenas), and **iii)** *Effect of transgene and mesocosm rearing:* T Mesocosm + NT Nature (n = 2 arenas). Behaviour is averaged over four daily 5 min daily intervals measured starting at 9 am, 11 am, 1 pm, and 3 pm. **A)** number of aggressive actions (chases and bites) given, **B)** number of aggressive actions received, **C)** numbers of times males attend females, **D)** number of times females are attended by males, **E)** number of times females dig or cover. Data are given as means over arenas ± standard error of the mean. Significant differences within trial type between groups/sex are indicated by letter (x,y) or by * on largest value, p<0.05.

*Effect of Transgene (NT Mesocosm + T Mesocosm):* NT Mesocosm and T Mesocosm fish did not differ in the number of aggressive actions given (p = 0.762) or received (p = 0.132), or in the number of attending behaviours given by males (p = 0.311) or received by females (p = 0.209, see Figures 7Ai–Di). However, the average time at which females received attending behaviour within the spawning trial differed, with T Mesocosm females receiving attention before NT Mesocosm females (48% and 62% through the spawning trial respectively, p = 0.022). NT Mesocosm and T Mesocosm fish did not differ significantly in number of quivers given (0.38±0.28 and 0.03±0.02 quivers/fish/5 min interval respectively, p = 0.343) or received (0.32±0.29 and 0.05±0.04 quivers received/fish/5 min interval respectively, p = 0.402), or in the number of digs and covers observed (p = 0.686, Figure 7Ei). There was a significant interaction between fish group and arena on aggressive actions given (p = 0.030), but no individual differences.

*Effect of Mesocosm Rearing Relative to Nature (NT Mesocosm + NT Nature)*: NT Mesocosm fish performed 13.1% of the number of aggressive behaviours performed by NT Nature fish (p = 0.015, Figure 7Aii), and received 1.7-fold more aggressive action than NT Nature fish (p = 0.041, Figure 7Bii). NT Mesocosm fish performed 36.7% of attending behaviour (p = 0.032, Figure 7Cii), and received 25.9% of the attending behaviour of NT Nature fish (p = 0.032, Figure 7Dii). NT Mesocosm fish performed 11.5% of the digs and covers that NT Nature fish performed (p = 0.029, Figure 7Eii), although the two groups of fish did not differ in number of quivers/gapes given (0.22±0.15 and 0.16±0.10 quivers+gapes/fish/5 min interval respectively, p = 0.751) or received (0.31±0.18 and 0.08±0.05 quivers+gapes received/fish/5 min interval respectively, p = 0.261).

*Effect of Transgene and Mesocosm Rearing (T Mesocosm + NT Nature)*: T Mesocosm fish performed only 31.6% of the aggressive behaviours of NT Nature fish (p<0.001, Figure 7Aiii), and received 2.3-fold more aggressive action than NT Nature fish (p = 0.023, Figure 7Biii). T Mesocosm males performed 32.7% of the attending behaviours of NT Nature males (p = 0.005, Figure 7Ciii). There was no difference between the two groups in the number of attending behaviours received by females (p = 0.333, Figure 7Diii), although NT Nature females received attending behaviours on average earlier than T Mesocosm females (at 37% and 69% of the way through the spawning trial respectively, p<0.001). NT Nature and T Mesocosm fish did not differ in number of quivers given (0.11±0.11 and 0.11±0.05 quivers/fish/5 min interval respectively, p = 0.663) or received (0.06±0.03 and 0.16±0.13 quivers received/fish/5 min interval respectively, p = 0.618), nor in numbers of digs and covers performed by females (p = 0.295, Figure 7Eiii). It should be noted that in one of the two T Mesocosm×NT Nature arenas, all NT Nature females had died by 72% of the way through the trial, leaving only T Mesocosm females to spawn and interact with. However, the only difference in spawning success and behaviour between the two arenas was that in the arena where all NT Nature females died early, no T Mesocosm females were observed digging, while in the other arena T Mesocosm fish had 2.4-fold the observed digging as NT Nature fish (0.295 and 0.125 digs/fish/5 min interval respectively), although they performed the digs later in the trial than NT Nature females (at 49% and 22% of the way through the spawning trial respectively).

*Effect of Mesocosm Rearing Relative to Standard Culture (NT Mesocosm + NT Culture)*: In the one arena examined, there were no differences between fish groups in number of aggressive actions given (p = 0.861) or received (p = 0.856), although male fish received more aggressive actions than female fish (p = 0.021, see Table 5 for all behaviour). NT Mesocosm males performed 2.2-fold more attending behaviours than NT Culture fish (p = 0.006). There was no difference between fish groups in the number of attending behaviours received (p = 0.729), although NT Mesocosm females received attention on average earlier than NT Culture females (p<0.001). NT Culture females performed more digs and covers than NT Mesocosm females (p = 0.003). There was no difference between fish groups in number of quivers given (p = 0.650), or received (p = 0.057), although NT Mesocosm females received quivers on average before NT Culture females (p<0.001).

**Table 5:** Courtship and spawning behaviour of NT Mesocosm and NT Culture fish in a Full Competition Spawning experiment.

Behaviour	NT Mesocosm ♀	NT Mesocosm ♂	NT Culture ♀	NT Culture ♂
*Number of aggressive behaviours* *given/fish/5* *min*	0.08±0.04	0.10±0.04	0.13±0.04	0.07±0.05
*Number of aggressive behaviours* *received/fish/5* *min*	0.04±0.02	0.14±0.04	0.05±0.03	0.14±0.05
*Number of attending behaviours* *given/male/5* *min*	n/a	0.54±0.08^a^	n/a	0.25±0.09^b^
*Number of attending behaviours* *received/female/5* *min*	0.32±0.08	n/a	0.54±0.19	n/a
*Average time of attending behaviours* *received as % of total time*	5.5±0.7%^a^	n/a	14.0±0.7^b^	n/a
*Number of digs/female/5* *min*	0^a^	n/a	0.26±0.11^b^	n/a
*Number of quivers given/male/5* *min*	n/a	0.21±0.09	n/a	0.31±0.18
*Number of quivers received* */female/5* *min*	0.08±0.05	n/a	0.43±0.19	n/a
*Average time quivers received* *as % of total time*	4.5±0.9%^a^	n/a	13.3±0.6^b^	n/a

NT = wild-type coho salmon, Mesocosm = fish were raised from smolt in a mesocosm, Culture = fish were raised from smolt in standard culture conditions. Four fish of each sex and fish group were represented in one arena. Behaviour is averaged over four 5 min daily intervals starting at 9 am, 11 am, 1 pm, and 3 pm during the spawning trial.

a,b indicates significant differences between fish groups, p<0.05.

#### IIc. Offspring Survival

Offspring survival (total offspring recorded as percent of expected offspring calculated from spawned egg mass) was estimated for the 2013-year class only, due to incomplete fecundity data for spawning females in other years. In this year, offspring from NT Mesocosm females had 5.1-fold greater survival than offspring from T Mesocosm females (50.4±12.0% and 9.9±2.9% respectively, p = 0.002). Offspring survival from NT Nature females 26.7±5.2%) did not differ significantly from either Mesocosm group in this year.

### III. High Male Competition Experiments for NT Nature Females

To specifically examine whether GH transgenesis and/or mesocosm rearing influences the spawning success of male salmon during competition, we compared the spawning success of T Mesocosm, NT Mesocosm, and NT Nature males (two types per trial) in competition for NT Nature females.

#### IIIa. Spawning Success

**Figure 8:**
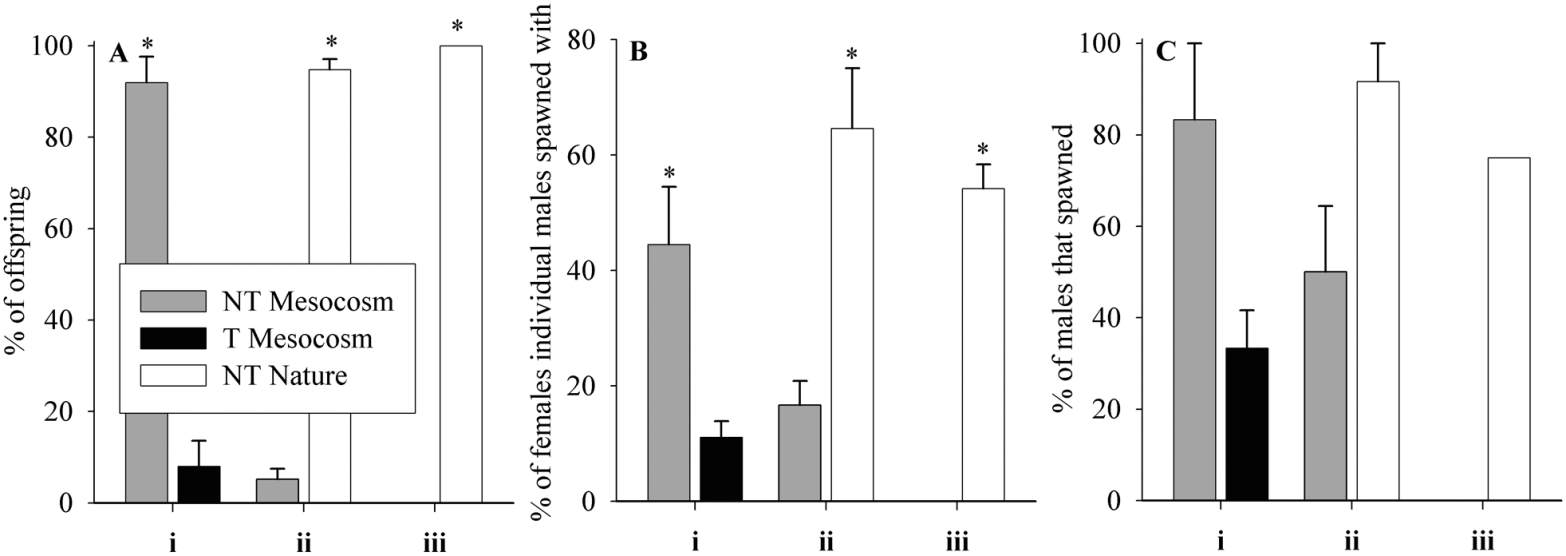
Spawning success of male fish groups during High Male Competition experiments for NT Nature females. Fish groups are wild-type (NT) and growth hormone transgenic (T) coho salmon raised in a Mesocosm or natural (Nature) conditions from smolt. Each spawning arena contained two groups of four male fish, in spawning competition for four NT Nature females (2∶1 Male:Female). Trials were **i)** *Effect of transgene:* NT Mesocosm ♂ vs T Mesocosm ♂ (n = 3 arenas), **ii)** *Effect of mesocosm rearing:* NT Mesocosm ♂ vs NT Nature ♂ (n = 3 arenas), and **iii)** *Effect of transgene and mesocosm rearing:* T Mesocosm ♂ vs NT Nature ♂ (n = 2 arenas). **A)** % of offspring male fish contributed to, **B)** % of available NT Nature female partners individual male fish spawned with, **C)** % of male individuals that spawned. Data are given as means over arenas ± standard error of the mean. Significant differences within trial type between groups/sex are indicated by * on the largest value, p<0.05.

*Effect of Transgene (NT Mesocosm ♂ vs T Mesocosm ♂):* NT Mesocosm males had 11-fold more offspring than T Mesocosm males in competition for NT Nature females (p<0.001, Figure 8Ai). As well, NT Mesocosm males had 4 times more partners on average than T Mesocosm males (p = 0.015, Figure 8Bi), but the two groups did not differ significantly in the % of males that spawned (p = 0.055, Figure 8Ci). There was a weak but significant correlation between fish length and number of offspring produced or proportion of available partners individual fish spawned with in T Mesocosm males only (p = 0.032 R^2^ = 0.38, and p = 0.043 R^2^ = 0.35 respectively).

*Effect of Mesocosm Rearing Relative to Nature (NT Mesocosm ♂ vs NT Nature ♂)*: NT Mesocosm males had 5.5% of the offspring that NT Nature males had in competition for NT Nature females (p<0.001, Figure 8Aii). As well, NT Mesocosm males spawned with 25.8% of the available females that NT Nature males spawned with (p = 0.027, Figure 8Bii), but did not differ significantly in the % of males that spawned (p = 0.200, Figure 8Cii). Neither offspring number nor percent of total available partners individual fish spawned with significantly correlated with fish length in any group (p = 0.363 to 0.972).

*Effect of Transgene and Mesocosm Rearing (T Mesocosm ♂ vs NT Nature ♂):* When NT Nature males were in competition with T Mesocosm males for NT Nature females, only NT Nature males successfully spawned (Figure 8Aiii–Ciii). Neither offspring number nor percent of total available partners individual fish spawned with significantly correlated with fish length (p = 0.338 and p = 0.274 respectively for NT Nature males).

#### IIIb. Behaviour

**Figure 9:**
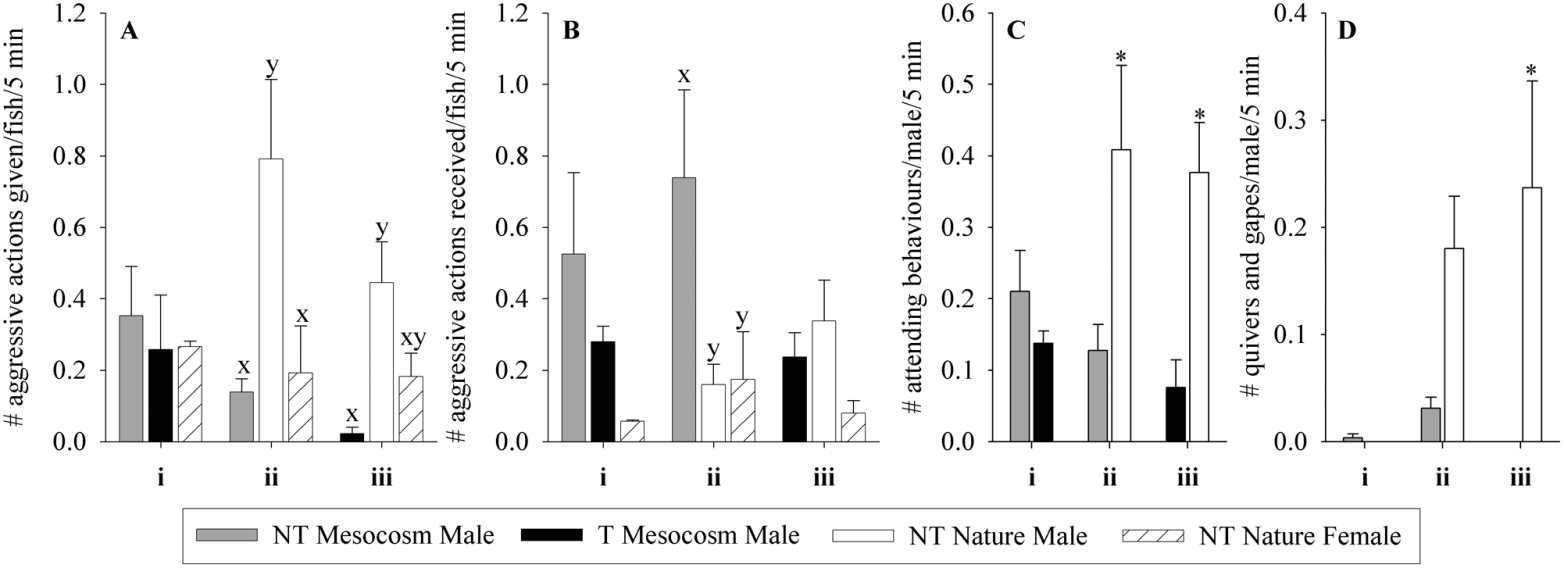
Spawning behaviour of male fish groups during High Male Competition experiments for NT Nature females. Fish groups are wild-type (NT) and growth hormone transgenic (T) coho salmon males raised in a Mesocosm or natural (Nature) conditions from smolt. Each arena contained two groups of four male fish in spawning competition for four NT Nature females in an artificial spawning arena. Trials were **i)** *Effect of transgene:* NT Mesocosm ♂ vs T Mesocosm ♂ (n = 3 arenas), **ii)** *Effect of mesocosm rearing:* NT Mesocosm ♂ vs NT Nature ♂ (n = 3 arenas), and **iii)** *Effect of transgene and mesocosm rearing:* T Mesocosm ♂ vs NT Nature ♂ (n = 2 arenas). Behaviour is averaged over four 5 min daily intervals measured starting at 9 am, 11 am, 1 pm, and 3 pm during the experiment. **A)** number of aggressive actions (chases and bites) given, **B)** number of aggressive actions received, **C)** numbers of times male attends NT Nature females, **D)** number of quivers given by male. Data are given as means over arenas ± standard error of the mean. Significant differences within trial type between groups/sex are indicated by letter (x,y) or by * over largest value, p<0.05.

*Effect of Transgene (NT Mesocosm ♂ vs T Mesocosm ♂):* NT Mesocosm and T Mesocosm males did not differ in overall number of aggressive actions given (p = 0.594) or received (p = 0.071), or in the number of attending behaviours (p = 0.087) or quivers (p = 0.317) given when in competition for NT Nature females (Figure 9Ai–Di). However, there was a significant interaction between fish group and arena in aggressive actions received (p = 0.046), where NT Mesocosm males received fewer aggressive actions than T Mesocosm males in one arena, but not in the other two arenas.

*Effect of Mesocosm Rearing Relative to Nature (NT Mesocosm ♂ vs NT Nature ♂)*: NT Mesocosm males performed only 17.5% of the aggressive actions that NT Nature males did (p<0.001, Figure 9Aii), while NT Mesocosm males received overall 4.6-fold more aggressive actions than NT Nature males (p<0.001, Figure 9Bii). However, there was a strong fish group×arena interaction for aggressive actions received (p = 0.002), where the greater aggressive actions received by NT Mesocosm males were only significant for one arena. NT Nature males performed 3 times the average number of the attending behaviours of NT Mesocosm males (p = 0.006, Figure 9Cii), although the two groups did not differ significantly in number of quivers given (p = 0.169, Figure 9Dii).

*Effect of Transgenic and Mesocosm Rearing (T Mesocosm ♂ vs NT Nature ♂):* T Mesocosm males performed only 5.1% of the aggressive actions that NT Nature males did (p<0.001, Figure 9Aiii), but did not differ significantly in number of aggressive actions received (p = 0.097, Figure 9Biii). T Mesocosm males also performed only 20.1% of the attending behaviours of NT Nature males (p = 0.001 Figure 9Ciii), and unlike NT Nature males they did not perform any observed quivers to NT Nature females (p<0.001, Figure 9Diii).

## Discussion

### Influence of Mesocosm Rearing on Spawning Success of Wild-type Fish

The spawning success and spawning behaviour (aggressive, courtship, and digging behaviours) of wild-type (NT) coho salmon raised in large (350,000 L), semi-natural seawater mesocosm approached that of nature-reared coho salmon under conditions without competition, but was much lower than nature-reared fish under competitive conditions (see Table 6 for an overall comparison of NT Mesocosm and NT Nature fish). NT Mesocosm fish displayed some of the spawning morphology of NT Nature fish, but were shorter in length, indicating mesocosm rearing only partially restored typical morphology of nature-reared spawning fish compared to rearing in smaller (4000 L) culture containers [23]. In competition trials (both Full Competition and High Male) comparing NT Mesocosm and NT Nature fish, NT Mesocosm fish had much lower spawning success and behaviour. This concurs with previous studies that found other types of culture-rearing reduced spawning behaviour and success in Pacific salmon [29]–[31]. In contrast, in experiments without inter-male competition (single male and female pairs), NT Mesocosm fish tended to have similar spawning success and behaviour as NT Nature fish.

**Table 6:** Summary of relative spawning success and behaviour of NT and T coho salmon reared in a semi-natural mesocosm or natural systems, in three different competition experiments (No, Full, and High Male Competition).

		Trials		
		NT Meso vs T Meso	NT Meso vs NT Nature	T Meso vs NT Nature
***OVERALL***		**≥**	**<**	**<**
*I. No Competition*				
a. Spawning success		=	≈	≈
b. Spawning behaviour		≈	≤	≤
*II. Full Competition*				
a. Spawning success		=	<	<
b. Spawning behaviour		=	<	<
*III. Male Competition*				
a. Spawning success		>	<	<
b. Spawning behaviour		=	<	<

NT = wild-type coho salmon. T = growth hormone transgenic coho salmon. Meso = reared in a semi-natural 350,000 L seawater tank from smolt. Nature = reared in natural systems from smolt. Spawning success = proportion of offspring contribute to, of individuals that spawned, and of mates spawned with. Spawning behaviour = aggressive, courtship (attending, quivers, gapes), and digging behaviours.

While rearing in a seawater mesocosm did not fully restore spawning success of wild-type fish, comparisons with previous experiments indicate that mesocosm rearing improved some aspects of spawning success and behaviour of wild-type fish over rearing in small culture tanks. In the one trial examined in the present study, NT Mesocosm males had greater spawning success and behaviour than NT Culture males, although NT Mesocosm females had slightly lower spawning success (and inconsistent relative behaviour) compared to NT Culture females. When results of the current Full Competition experiments were compared with that of culture-reared wild-type fish in Fitzpatrick et al. [24], mesocosm-reared fish had better spawning success than culture-reared fish when in competition with NT Nature fish (25% and 13% of nature-reared fish respectively), although had similar patterns of suppressed spawning and courtship behaviour. The present study showed no significant differences in paired (No Competition) spawning success between wild-type mesocosm-reared fish and nature-reared fish, while Bessey et al. [23] found culture-reared wild-type females had lower spawning success than full nature-reared pairs, although the patterns of spawning success were similar between the two studies. During spawning, patterns of quivers surrounding a spawning event differed between the current study and Bessey et al. [23], where only mesocosm-raised wild-type fish shared the pattern displayed by nature-reared fish of increasing quivers up to a spawning event, then decreasing quivers post-spawning [54]. Overall, seawater mesocosm rearing appeared to increase spawning success of wild-type coho over standard culture conditions while in competition with nature-reared fish, but improvements in spawning success in No Competition experiments are less clear. The factors associated with mesocosm culture that prevent full restoration of spawning success and behaviour are not known, but likely include lack of habitat complexity and limited spatial scope in the mesocosm environment. As well, diet differences between mesocosm- and nature-reared fish (commercial versus natural) may have influenced spawning colouration, and consequent attractiveness to potential mates. In Full Competition experiments with NT Mesocosm fish, spawning success was significantly correlated to body length in NT Nature fish. While this correlation was not observed in NT Mesocosm fish, the smaller length of NT Mesocosm fish may have decreased their mating advantage with NT Nature fish. This would concur with other studies in Pacific salmon where size has been reported to have significant impact on mating advantage in male and, to a lesser extent, female fish [26], [28].

### Spawning Success of Mesocosm-reared GH Transgenic Salmon

As observed in other GH transgenic salmon studies [23]–[25], T Mesocosm fish had very low reproductive success relative to wild-type salmon reared in nature. However, when compared to wild-type fish grown in the mesocosm, T Mesocosm fish had equal spawning success in Full Competition and No Competition experiments, and displayed similar overall spawning behaviour (see Table 6 for an overall comparison of T Mesocosm and NT fish). In Full Competition, this held true whether T and NT Mesocosm fish were in direct competition, or in competition with NT Nature fish. This concurs with studies in other fish models, where GH transgenic catfish, medaka, and carp had similar reproductive success as wild-type fish grown in equal conditions [22], [32], [33]. In contrast, other studies found GH transgenic Atlantic salmon parr, zebrafish, and medaka had lower reproductive success compared to wild-type fish grown in equal conditions [25], [34]–[36], although GH transgenic medaka males have inconsistently higher reported mating success compared to wild-type [11]. There was no evidence for assortative mating of T Mesocosm and NT fish in any trial examined. In the present study, T Mesocosm fish differed from NT Mesocosm fish in a few ways that could indicate this strain of GH transgenic fish, particularly males, have decreased spawning success in some circumstances. NT Nature females chose NT Mesocosm males over T Mesocosm males in High Male Competition, indicating differences between wild-type and transgenic fish were present that were of significance to mate choice of NT Nature fish or to spawning success of males. As well, during High Male Competition with NT Nature males, T Mesocosm males failed to spawn, were not observed to quiver, and participated in much fewer aggressive behaviours than NT Mesocosm males in competition with NT Nature males. While behaviour of T Mesocosm and NT Mesocosm males in the High Male Competition experiments did not significantly differ, T Mesocosm fish in general did deviate more from typical spawning morphology than NT Mesocosm fish, which could have played a role in mate choice by NT Nature females, and/or competitive ability of T fish. The excessive cranial growth in some transgenic strains under standard culture conditions [23], [55], [56] is not, prior to maturation, pronounced in the strain under study (M77 strain), however these abnormalities became apparent in some T mesocosm fish at maturation. In addition, the extreme deep body of some T Mesocosm fish in the 2010 year has not been previously observed (and curiously was seen only in one year in the present studies), but such changes in overall body proportion would likely influence reproductive success compared to a wild phenotype. Despite their larger size, T Mesocosm fish did not have significantly greater spawning success than NT Mesocosm fish indicating size alone does not influence spawning success in coho, although larger T fish tended to have greater spawning success in competition than smaller T fish. As well, transgenic coho salmon reared in standard culture have lower ejaculate density and sperm mobility [24], potentially exacerbating the lower spawning ability of male transgenic fish. The greater impact of GH transgenesis on spawning success of male versus female salmon concurs with other salmonid models where spawning success of male fish was more suppressed by hatchery rearing, or domestication combined with culture-rearing, than in female fish [26], [27], [39]–[41], likely due to a more intense breeding competition in male relative to female salmonids (see [57]). As well, transgenic fish used in the High Male Competition experiments were ration restricted during juvenile growth. In GH transgenic medaka, males had lower relative spawning success after rearing on low versus high food availability [36]. As such, poor reproductive performance of T Mesocosm males in the High Male Competition experiments may have been exacerbated by restricted juvenile growth in these fish.

When NT and T Mesocosm fish were in full competition, there were a number of transgenic females that appeared to spawn some of their egg mass, but did not contribute to the final offspring population. Offspring survival as percent of expected eggs spawned was much lower in T Mesocosm females than NT Mesocosm females. As such, T Mesocosm females appear to have increased incidence of unfertilized eggs and/or egg mortality. This could be due to unsynchronized dropping of eggs and milt in a spawning pair, or nest destruction due to poorly covered eggs. During a spawning event in No Competition experiments, T Mesocosm males did not have a typical pattern of quivers up to and after a spawning event. This may have interfered with synchronization of spawning events, although T Mesocosm fish did not differ from NT Mesocosm fish in the percent of spawning events with visible eggs and/or milt in No Competition experiments. As well, T Mesocosm females covered less than NT fish immediately after a spawning event. In Full Competition experiments, spawning areas provided were 1.92 m^2^/spawning female, which is 30% less than the reported average nest size for coho salmon in nature (2.8 m^2^/female, [43]). As well, we found most nests were located in the downstream section of the arenas with several nests superimposed and overlapping. The poor covering by T Mesocosm fish (as expressed by low number of digs immediately after spawning) may indicate their nests were more susceptible to damage in such a highly competitive arena, resulting in decreased survival of offspring. In addition, differential mortality of T offspring has been noted in different strains of GH transgenic coho [58] and well as in GH transgenic carp [33], which could result in overall lower survival of transgenic female offspring. Transgenic fish tend to have higher fecundity in coho salmon and medaka [22], [23], but potential for poor nest covering, unsynchronized spawning, and differential mortality of transgenic offspring may offset this advantage. Both NT and T Mesocosm females retained more eggs than NT Nature females (assessed at death post-spawning), and several mesocosm-raised individuals did not spawn at all. This concurs with Berejikian et al. [31] who first noted such effects, finding captive-reared wild-strain chinook salmon deposited 50% fewer eggs than nature-reared females, and many captive-reared fish did not participate in spawning events.

### Genotype-by-Environmental Interactions on Spawning Success

**Figure 10:**
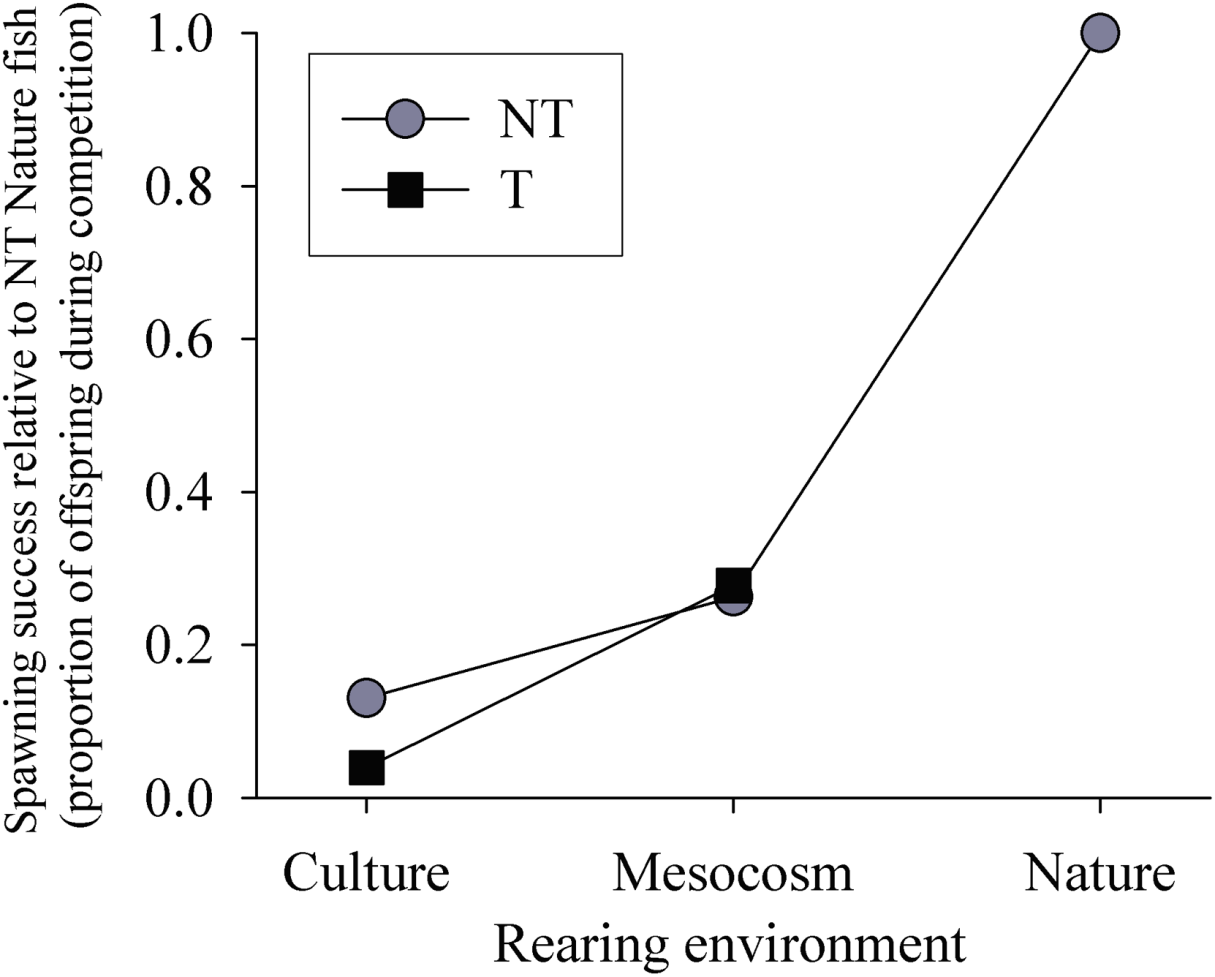
Genotype×Environmental reaction norms of spawning success of coho salmon in competition. Genotypes are wild-type (NT) and growth hormone transgenic (T) coho salmon reared in environments of standard Culture (4000 L tank, data taken from [24]), a semi-natural Mesocosm (350,000 L), or natural environments (Nature, NT only) from smolt. Spawning success is given as proportion of offspring fish group contributed to when in full competition (mixed male and female fish) with NT Nature fish, relative to the success of NT Nature fish. The lack of parallel reaction norms of NT and T fish between culture and mesocosm environments, and the unknown slope of the reaction norm for T fish between Mesocosm and Nature complicates predictions of spawning success of T fish in nature.

The spawning success of GH transgenic coho salmon relative to equally reared wild-type fish appears to be influenced by rearing background, as well as type of spawning trial. In full competition, GH transgenic fish raised in standard culture had only 30% of the spawning success of equally-raised wild-type fish as measured by percent of offspring [24], but T Mesocosm and NT Mesocosm fish had approximately equal spawning success in competition with NT Nature fish in the current study (see Figure 10 for reaction norms of spawning success in competition). This suggests the relative spawning success of GH transgenic and wild-type fish can be influenced by the environmental conditions of seawater rearing and or experimental assessment. One difference between the current study and Fitzpatrick et al. [24] is spawning competition was examined under lower spawning female density (1.92 m^2^/female versus 1.13 m^2^/female, respectively). As such, the greater success of T Mesocosm fish relative to culture-raised transgenic fish may be due in part to transgenic fish performing poorly in the more competitive environment of Fitzpatrick et al. [24], rather than entirely from benefits due to mesocosm rearing.

In male competition, Bessey et al. [23] found wild-type and transgenic culture-reared males had equally poor spawning success when in direct competition for a single nature-reared female, while the current study showed NT Mesocosm males greatly outcompeted T Mesocosm males in competition for NT Nature females. This indicates that wild-type males benefited more from mesocosm rearing over standard culture than did transgenic males, in contrast to the results of the Full Competition experiments. These effects also may be due to differences in spawning trial (2 males to 1 female in [23], versus 8 males to 4 females in the current study). As well, the transgenic males used in the current High Male Competition experiments were ration restricted during freshwater growth, while previous studies and the Full Competition experiments in the current study used transgenic fish that were fully-fed during juvenile rearing, which may have influenced their spawning ability (see above).

In No Competition experiments, while there were no significant differences in spawning success of NT Nature, NT Mesocosm or T Mesocosm fish, the general trends of spawning success followed that reported in Bessey et al. [23]. One exception to this is that transgenic females raised in culture had low spawning success with nature-reared males (12.5% spawned; [23]), while in the current study, mesocosm-reared transgenic females had medium spawning success with nature-reared males (50%), suggesting GH transgenic females may have greater spawning success without competition if raised in a mesocosm relative to standard culture conditions. The presence of genotype-by-environmental interactions on spawning success of GH transgenic fish concurs with Pennington and Kapuscinski [36] who found interacting influences of food availability and predator presence on spawning success of GH transgenic medaka. As such, extrapolating existing spawning data of GH transgenic fish to new environments (e.g. nature) is complicated by the non-parallel reaction norms seen for reproductive success among different environmental conditions.

## Conclusions

Overall, mesocosm rearing from smolt to adulthood only partially restored spawning success to wild-type coho salmon, but critically did increase their reproductive capabilities to a level where relative comparisons to GH transgenic salmon can be made. Among all experiments conducted to date with strain M77 GH transgenic and reference wild-type coho salmon reared and studied under a range of environmental conditions, the data does not support this strain of GH transgenic fish possessing any mating advantage over wild-type. Under some conditions GH transgenic salmon have reduced reproductive success, and overall it is possible, but not certain, their fitness would be lower than that of wild-type. From an evolutionary perspective, wild-type salmon, which have significant capacity to select for different growth rates, have adjusted growth to maximize fitness under natural conditions, and it is unlikely that an anthropogenically-induced adjustment to such a phenotype would result in an enhancement of fitness for the animal in its present niche. However, it is also possible that quantum shift in growth and behaviour caused by GH transgenesis could provide access to a phenotype not accessible to wild strains (due to strong balancing selection), or could cause effects on other traits (e.g. feeding competition) that provide compensating benefits to net fitness [22]. While theoretical considerations are critical, determination of fitness effects of GH transgenesis and environmental influences on them need to be empirically determined for assessment of actual risk. Indeed, previous and current studies indicate significant genotype-by-environmental interactions exist, where the type of seawater rearing appears to exert differing influences on the spawning success of wild-type and GH transgenic coho salmon (see Figure 10). The relative spawning success of wild-type and transgenic fish was also greatly influenced by the type of spawning study, indicating care must be taken when extrapolating spawning success from a single study type. Together, existing data indicate escaped GH transgenic salmon would be capable of reproducing in the wild and successfully spawning with wild populations, although the extent to which they may do this is not fully known. If mesocosm rearing had restored the reproductive success of wild-type coho salmon to that seen for wild salmon from nature, we may have felt more confident that the reproductive success of transgenic salmon reared in the same conditions would more closely approximate that for the same strain should it be derived from nature. Thus, until we have improved ability to predict the phenotype of transgenic salmon in nature, insufficient data exists to extrapolate with low uncertainty what the true spawning success, and hence risk, of these fish would be should they escape early in life and spend smolt to adulthood in natural conditions. Given the scale and scope of the present experiments, overcoming these limitations in empirical assessment will be challenging.
